# Metabolomic analysis of obesity, metabolic syndrome, and type 2 diabetes: amino acid and acylcarnitine levels change along a spectrum of metabolic wellness

**DOI:** 10.7717/peerj.5410

**Published:** 2018-08-31

**Authors:** Diane M. Libert, Amy S. Nowacki, Marvin R. Natowicz

**Affiliations:** 1 Cleveland Clinic Lerner College of Medicine of Case Western Reserve University, Case Western Reserve University School of Medicine, Cleveland, OH, United States of America; 2 Department of Quantitative Health Sciences, Cleveland Clinic, Cleveland, OH, United States of America; 3 Pathology and Laboratory Medicine, Genomic Medicine, Pediatrics and Neurological Institutes, Cleveland Clinic, Cleveland, OH, United States of America

**Keywords:** Metabolomic, Metabolomics, Obesity, Metabolic syndrome, Metabolic wellness, Type 2 diabetes, Amino acids, Acylcarnitines

## Abstract

**Background:**

Metabolic syndrome (MS) is a construct used to separate “healthy” from “unhealthy” obese patients, and is a major risk factor for type 2 diabetes (T2D) and cardiovascular disease. There is controversy over whether obese “metabolically well” persons have a higher morbidity and mortality than lean counterparts, suggesting that MS criteria do not completely describe physiologic risk factors or consequences of obesity. We hypothesized that metabolomic analysis of plasma would distinguish obese individuals with and without MS and T2D along a spectrum of obesity-associated metabolic derangements, supporting metabolomic analysis as a tool for a more detailed assessment of metabolic wellness than currently used MS criteria.

**Methods:**

Fasting plasma samples from 90 adults were assigned to groups based on BMI and ATP III criteria for MS: (1) lean metabolically well (LMW; *n* = 24); (2) obese metabolically well (OBMW; *n* = 26); (3) obese metabolically unwell (OBMUW; *n* = 20); and (4) obese metabolically unwell with T2D (OBDM; *n* = 20). Forty-one amino acids/dipeptides, 33 acylcarnitines and 21 ratios were measured. Obesity and T2D effects were analyzed by Wilcoxon rank-sum tests comparing obese nondiabetics vs LMW, and OBDM vs nondiabetics, respectively. Metabolic unwellness was analyzed by Jonckheere-Terpstra trend tests, assuming worsening health from LMW → OBMW → OBMUW. To adjust for multiple comparisons, statistical significance was set at *p* < 0.005. K-means cluster analysis of aggregated amino acid and acylcarnitine data was also performed.

**Results:**

Analytes and ratios significantly increasing in obesity, T2D, and with worsening health include: branched-chain amino acids (BCAAs), cystine, alpha-aminoadipic acid, phenylalanine, leucine + lysine, and short-chain acylcarnitines/total carnitines. Tyrosine, alanine and propionylcarnitine increase with obesity and metabolic unwellness. Asparagine and the tryptophan/large neutral amino acid ratio decrease with T2D and metabolic unwellness. Malonylcarnitine decreases in obesity and 3-OHbutyrylcarnitine increases in T2D; neither correlates with unwellness. Cluster analysis did not separate subjects into discreet groups based on metabolic wellness.

**Discussion:**

Levels of 15 species and metabolite ratios trend significantly with worsening metabolic health; some are newly recognized. BCAAs, aromatic amino acids, lysine, and its metabolite, alpha-aminoadipate, increase with worsening health. The lysine pathway is distinct from BCAA metabolism, indicating that biochemical derangements associated with MS involve pathways besides those affected by BCAAs. Even those considered “obese, metabolically well” had metabolite levels which significantly trended towards those found in obese diabetics. Overall, this analysis yields a more granular view of metabolic wellness than the sole use of cardiometabolic MS parameters. This, in turn, suggests the possible utility of plasma metabolomic analysis for research and public health applications.

## Introduction

Obesity, a major risk factor for cardiovascular disease, type 2 diabetes (T2D), and all-cause mortality, has dramatically increased in population prevalence over the past several decades (Ogden, Carroll & Flegal, 2003; The GBD 2015 Obesity Collaborators, 2017). Besides obesity, a constellation of factors is associated with the development of cardiovascular disease, T2D, and all-cause mortality. To group these factors into standardized criteria, four main definitions of metabolic syndrome (MS) have been formulated: the original World Health Organization (WHO) definition of 1998; criteria of the European Group for the study of Insulin Resistance (EGIR); criteria of the National Cholesterol Education Program Adult Treatment Panel (NCEP ATP) III; and criteria of the International Diabetes Foundation (IDF) (Alberti & Zimmet, 1998; Balkau & Charles, 1999; Zimmet et al., 2005; Grundy et al., 2005). These criteria differ, but most include increased body mass index, hyperglycemia, hypertension, and dyslipidemia to describe an individual’s degree of progression toward cardiovascular disease and/or T2D. These criteria provide useful clinical and pathophysiological insights. However, the use of these criteria has also led to the recognition of a population of obese individuals who have been designated as “metabolically well,” and studies differ over whether this group has a higher morbidity and earlier mortality than their lean counterparts (Fan et al., 2013; Kramer, Zinman & Retnakaran, 2013; Mørkedal et al., 2014; Lavie, De Schutter & Milani, 2015).

This controversy suggests that current definitions of MS do not completely describe physiologic risk factors or consequences of this process. To assess this, there is increased interest in discovering biomarkers that differentiate those who are obese and metabolically well from those who are obese and metabolically unwell (Zhong et al., 2017). Metabolic signatures of obesity and diabetes have previously been studied to gain insight into the pathophysiology of these conditions and to develop and evaluate treatments; plasma amino acids and acylcarnitines are frequently assessed for these purposes. A large body of research characterizes metabolic differences in obese and diabetic states (Table S1). Similar studies on MS exist, but the definitions of MS and populations studied vary (Table S2). Elevations in the branched-chain amino acids (BCAAs; leucine, isoleucine, valine) and aromatic amino acids (AAAs; phenylalanine, tyrosine) are often reported in these populations (Felig, Marliss & Cahill, 1969; Jeevanandam, Ramias & Schiller, 1991; Newgard et al., 2009; Suhre et al., 2010; Xu et al., 2013). Studies of differences in acylcarnitine levels between obese or diabetic individuals and normal controls have been less consistent, but generally report increased levels of the BCAA-associated species (i.e., propionylcarnitine, butyrylcarnitine, isovalerylcarnitine) (Adams et al., 2009; Mihalik et al., 2012; Rauschert et al., 2014; Schooneman et al., 2014). Less consistent results were obtained for other amino acids or acylcarnitines. The differences between these studies may be attributable to variations in study populations or analytic methods.

To our knowledge, there are no data regarding metabolite levels in different populations along a spectrum of metabolic wellness from lean, metabolically well individuals to those who are obese and metabolically unwell with T2D. Using this approach, we sought to evaluate differences in the levels of plasma amino acids and acylcarnitines between lean healthy control adults, persons with obesity who are metabolically well by ATP III criteria, persons with obesity who meet criteria for metabolic syndrome, and obese individuals who are metabolically unwell and have T2D, thereby providing insights regarding the pathophysiological mechanisms associated with these states as well as their possible utility in the clinic. We anticipated that differences in amino acid and acylcarnitine levels would provide a more granular view of metabolic wellness than traditional MS criteria.

## Materials and Methods

### Study populations

Leftover plasma samples were collected from patients aged 20–59 years presenting to our hospital for outpatient fasting lipid panels and plasma metabolic panels. All patients had fasted 10–12 h. Ninety samples were collected on a continuous basis between July and October 2016 and stored at 4 °C for one to seven days in lithium heparin tubes before being decanted and frozen at −14 °C for short-term storage (<2 months) and −70 °C for long-term storage. Demographic data, medications, and laboratory test results were collected from the electronic medical record before the samples were de-identified and stored. The Institutional Review Board of the Cleveland Clinic granted approval to carry out the study (IRB #16-499).

### Determination of metabolic wellness

Metabolic wellness was determined according to the Adult Treatment Panel III (ATP III) guidelines for the clinical identification of metabolic syndrome; determinants of unwellness included blood pressure (≥130/≥85 mm Hg), fasting glucose (≥110 mg/dL), HDL (<40 mg/dL for men, <50 mg/dL for women), triglycerides (≥150 mg/dL), and waist circumference (>40 in. for men, >35 in. for women) (Grundy et al., 2005). Metabolically unwell individuals meet three or more criteria. Since waist circumference data were unavailable, we defined metabolically well individuals as those with at most one ATP III criterion and metabolically unwell individuals as having three or more criteria. Our four study groups were defined as follows:

• Lean metabolically well (LMW): BMI <25 kg/m^2^ with ≤1 ATP III criterion
• Obese metabolically well (OBMW): BMI >30 kg/m^2^ with ≤1 ATP III criterion
• Obese metabolically unwell (OBMUW): BMI >30 kg/m^2^ with ≥ 3 ATP III criteria
• Obese metabolically unwell, diabetic (OBDM): BMI >30 kg/m^2^ with ≥3 ATP III criteria and a documented diagnosis of type 2 diabetes.

### Exclusion criteria

To exclude patients with common or severe metabolic derangements other than metabolic syndrome or diabetes, we excluded persons having: (1) elevated creatinine (>1.00 mg/dL) or decreased eGFR (<60 mL/min/1.73 m^2^); (2) elevated liver enzymes (ALT >45 U/L, AST >40 U/L, alkaline phosphatase >150 U/L) or a diagnosis of liver disease besides nonalcoholic steatohepatitis; (3) abnormal plasma calcium levels; (4) plasma electrolyte disturbances; and (5) abnormal free T4 (<0.9 or >1.7 ng/dL) or abnormal TSH (>5.5 or <0.4 uU/mL) levels. We also excluded individuals with uncontrolled bipolar, unipolar, and major depressive disorder, persons with cancer or known genetic metabolic disorders, persons with acute inflammatory processes, and hospital in-patients.

### Metabolite quantification

High-performance liquid chromatography with ultraviolet detection (HPLC-UV) was used to quantify 41 plasma amino acids and related compounds (Narayan et al., 2011). Liquid chromatography-tandem mass spectrometry (LC-MS/MS) was used to measure free carnitine and 33 acylcarnitine species using a modification of the method of Scott, Heese & Garg (2016). Homocysteine was measured by a chemiluminescense immunoassay (Tewari, Zhang & Bluestein, 2004). Medians of the absolute values of these metabolites and calculated ratios for each group are reported in Tables S5A–S5C. Insulin levels were not measured.

### Storage and sensitivity analyses

To evaluate the effect of storage at 4 °C on our metabolite measurements, we first assessed possible changes of metabolite levels over time using blood from two healthy, fasting adults. Samples were obtained in lithium heparin tubes, centrifuged at 2500 rpm for 10 min in a Silencer 2310R centrifuge and analyzed immediately (baseline) and after storage at 4 °C for one, two, three, five, and seven days. The day at which levels deviated from baseline by 20% were recorded. It is recognized that length of storage could impact quality of the specimen. Therefore, a sensitivity analysis was performed where only samples stored at 4 °C for less than three days were analyzed. Results were consistent with those based on all samples and are reported in the supplementary materials (Tables S3A–S3C).

### Statistical analyses

Summary measures are reported as median (Q_1_–Q_3_), mean (standard deviation) or count (%) as appropriate. Differences in metabolite levels among the four groups were initially analyzed using Kruskal-Wallis tests. Acknowledging the large number of comparisons being performed, a significance level of *p* < 0.005 was utilized to reduce the type I error rate. Metabolites signifying variations among the four groups were further explored to better understand the differences. Specifically, three a priori contrasts of interest were investigated comparing: (1) obese non-diabetic (OBMW and OBMUW) to non-obese (LMW); (2) the metabolic wellness trend among non-diabetic patients (assuming worsening metabolic wellness from LMW → OBMW → OBMUW); and (3) diabetic (OBDM) to non-diabetic patients (LMW, OBMW, and OBMUW). The obesity and diabetes effects were assessed using nonparametric Wilcoxon rank-sum tests. The metabolic wellness trend was assessed using nonparametric Jonckheere-Terpstra trend tests. A calculation was conducted for a Wilcoxon rank-sum test to determine the power achieved for samples of size *n* = 20, 22 and 26 using an alpha of 0.005, a *t*_*n*−2_ distribution, a location difference of 1.25, and two tails resulting in 75%, 82% and 90% power respectively.

An unsupervised cluster analysis was performed to assess whether metabolic groups would form organically. A clustering task consists of dividing a dataset into groups (i.e., clusters) that share common properties according to given criteria and similarity metrics (Bailey, 1994). We employed the K-means algorithm (Jain, 2010) which relies on the frequent computation of similarity metrics between all of the elements to be clustered and the proposed centroids of each of the k-clusters. All analyses were performed using either JMP Pro version 13.0 or SAS statistical software version 9.4 (SAS Institute, Cary, NC, USA).

## Results and Discussion

### Demographic and clinical characteristics of the study populations

Plasma samples from 24 LMW, 26 OBMW, 20 OBMUW, and 20 OBDM subjects were analyzed. Obesity was regarded as BMI >30 kg/m^2^ and metabolic wellness was determined by ATP III criteria as described in the Methods section, ‘Determination of metabolic wellness’. Subjects were between 20 and 59 years of age with mean ages 36.8 (LMW), 37.5 (OBMW), 38.7 (OBMUW), and 40.0 (OBDM) (Table 1). Approximately half of the subjects in each group were female; 63% of LMW, 54% of OBMW, 45% of OBMUW, and 50% of OBDM. Additional demographic and clinical information are reported in Table 1. BMI, systolic and diastolic blood pressures, triglycerides, and fasting glucose level increased with the progression of metabolic unwellness from LMW to OBDM, while median HDL levels decreased. Such trends are expected as these factors were used to categorize the subjects.

**Table 1:** Demographics and clinical characteristics. Values are presented as Median (*Q*_1_, *Q*_3_) unless indicated otherwise. Units are reported as (mg/dL) unless otherwise indicated. eGFR values were calculated using the MDRD equation. Each patient taking a blood pressure, lipid, or diabetes medication was taking at least one but may be taking multiple medications.

	LMW	OBMW	OBMUW	OBDM
Total N	24	26	20	20
Patient Age-Mean (SD)	36.8 (12.4)	37.5 (10.5)	38.7 (11.7)	40.0 (10.8)
Female-no. (%)	15 (63)	14 (54)	9 (45)	10 (50)
Race-no. (%)				
White	19 (79)	16 (61)	15 (75)	16 (80)
Black	0 (0)	7 (27)	1 (5)	3 (15)
Other or Unknown	5 (21)	3 (12)	4 (20)	1 (5)
Smokers-no. (%)	2 (8)	3 (12)	2 (10)	2 (10)
BMI (kg/m^2^)	23.1 (21.6, 24.3)	35.0 (33.0, 39.7)	37.4 (32.6, 40.0)	41.3 (34.3, 47.0)
Diastolic BP (mm Hg)	68 (60.8, 78)	76.5 (69.5, 82.5)	89.5 (86, 96.8)	84 (70, 88)
Systolic BP (mm Hg)	115 (109, 120)	122 (112, 125)	136 (131, 140)	130 (121, 138)
Triglycerides	81 (59, 98)	86 (68, 113)	228 (194, 356)	227 (187, 288)
Total Cholesterol	172 (152, 200)	193 (164, 203)	208.5 (182, 242)	178 (151, 200)
HDL	60 (47, 68)	50 (39, 63)	34 (31, 40)	36 (32, 37.8)
VLDL	16.5 (11.5, 19.8)	17 (13.8, 22.3)	45.5 (39, 56.8)	45 (37.3, 57)
LDL	100 (77, 119)	114 (95, 131)	127 (104, 151)	98 (74, 115)
Glucose	85 (82, 90)	87 (84, 99)	100 (92, 107)	147 (130, 229)
Creatinine	0.87 (0.78, 1.02)	0.85 (0.77, 1.00)	0.87 (0.80, 1.02)	0.82 (0.70, 0.95)
eGFR (mL/min/1.73 m^2^)	81.3 (76.9, 88.0)	85.3 (79.1, 106.5)	88.2 (75.1, 98.5)	87.5 (79.5, 112.0)
Blood urea nitrogen	13 (10, 15)	12(11,14)	13(11,17)	13(11,15)
BP meds-no. (%)	0 (0)	2 (8)	5 (25)	4 (20)
Lipid meds-no. (%)	1 (4)	2 (8)	4 (20)	6 (30)
Oral DM meds-no. (%)	0 (0)	2 (8)	1 (5)	11 (55)
Insulin-no. (%)	0 (0)	0(0)	0(0)	3(15)

**Notes.**

Abbreviations BMI body mass index BP blood pressure eGFR estimated glomerular filtration rate HDL high density lipoprotein LDL low density lipoprotein SD standard deviation VLDL very low density lipoprotein

### BCAAs and related metabolites are elevated in obese, metabolically unwell, and diabetic states

**Figure 1:**
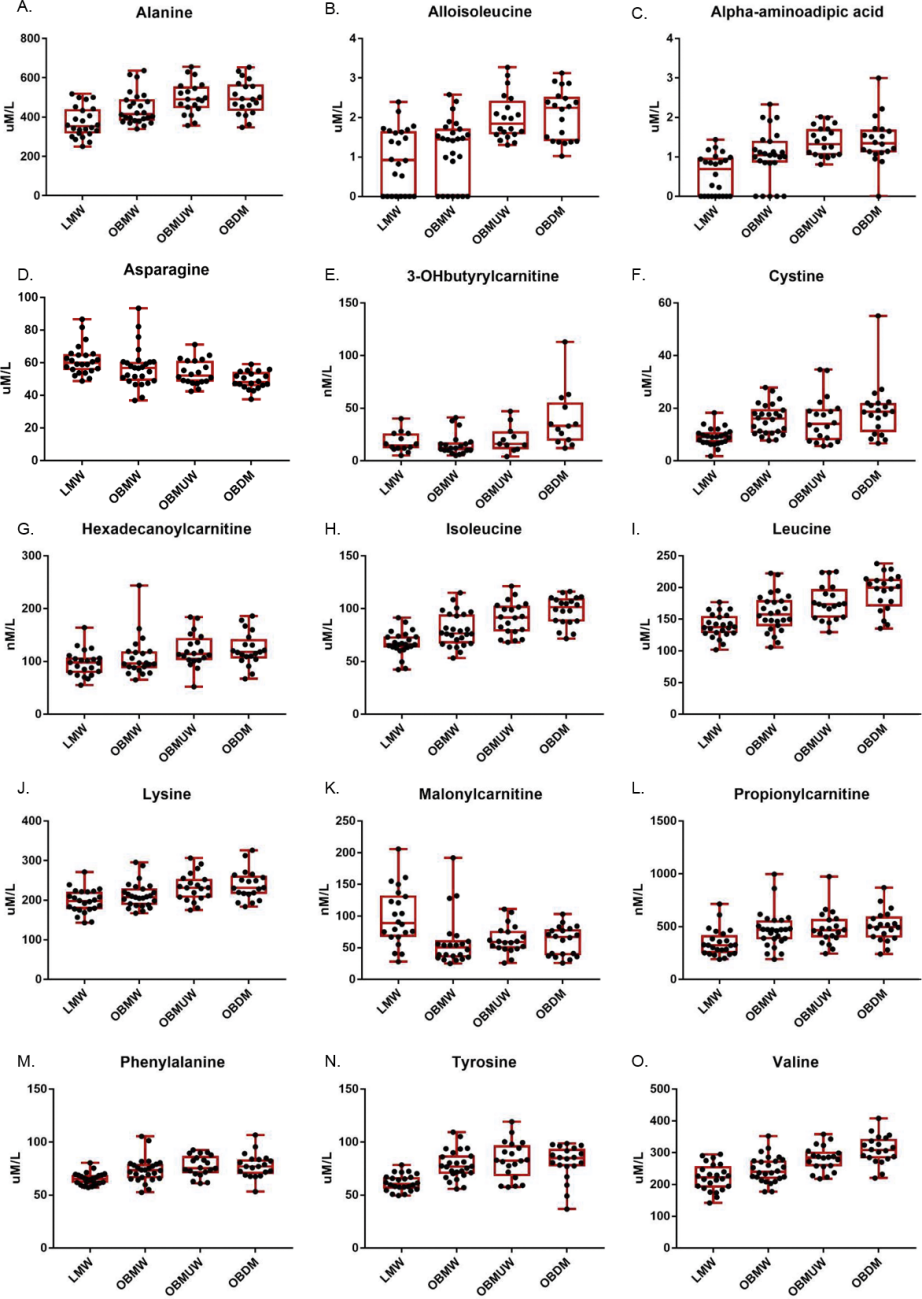
Box and whisker plots of plasma amino acid and acylcarnitine species with significant group differences (Kruskal-Wallis test, *p* < 0.005). The subject number in each group was as follows: LMW = 24, OBMW = 26, OBMUW = 20, OBDM = 20 except in the case of 3-OHbutyrylcarnitine, which was specifically quantified in 58 subjects (LMW = 14, OBMW = 20, OBMUW = 11, OBDM = 13). The species quantified in the other subjects could be either 3-OHisobutyrylcarnitine or 3-OHbutyrylcarnitine. Acylcarnitine levels are reported as nanomoles/liter, except for free carnitine and acetylcarnitine that are reported as micromoles/liter.

Our data indicate an association of increased levels of the BCAAs with diabetes, obesity and metabolic unwellness. Valine, leucine and isoleucine—all essential amino acids—are associated with all three states with strong statistical significance (*p* < 0.0001 overall for each amino acid, see Table 2 and Fig. 1 for specifics). These findings have been noted in other studies (Tables S1, S2). Alloisoleucine, formed from the racemization of isoleucine and its subsequent retransamination, is increased in metabolic unwellness (*p* = 0.0001) and diabetes (*p* = 0.004) (Table 2, Fig. 1). This finding is previously unreported but consistent with the elevated levels of isoleucine that are observed in these states (Table 2). Propionylcarnitine, a metabolite of valine and isoleucine metabolism, is increased in obese (*p* = 0.0003) and metabolically unwell states (*p* = 0.0006) (Table 2, Fig. 1). This has been found in several studies (Table S1) but, interestingly, two studies reported decreased propionylcarnitine in obese African American females with T2D (Adams et al., 2009; Fiehn et al., 2010). The latter points to the potential relevance of race in the investigation of acylcarnitines and other biomarkers in obesity and diabetes as well as the need for careful matching of cases and controls. We also observed an increased ratio of short chain acylcarnitines to all carnitine species in obesity (*p* = 0.0004), metabolic unwellness (*p* = 0.004) and diabetes (*p* = 0.0007) (Table 3), consistent with the increased levels of the BCAAs in these states and the metabolic derivation of these acylcarnitines; this has not been previously reported.

**Table 2:** *P*-values reflecting overall group differences, the obesity effect, metabolic unwellness trend, and diabetic effect. *P*-values < 0.005 were considered statistically significant and are bolded. Trend tests identified increasing (↑) or decreasing (↓) trends from LMW to OBMW to OBMUW groups. Group comparisons identified increasing (↑) or decreasing (↓) if the second listed group had higher or lower values, respectively. For example, Obesity Effect for malonylcarnitine 0.0003 (↓) means that OBMW & OBMUW subjects have lower levels than LMW subjects.

Species	Overall	Obesity effect (LMW vs. OBMW, OBMUW)	Metabolic Wellness Trend (Nondiabetic) (LMW → OBMW → OBMUW)	Diabetic effect (LMW, OBMW, OBMUW vs. OBDM)
Alanine	**<0.0001**	**<0.0001 (↑)**	**<0.0001 (↑)**	0.008
Alloisoleucine	**<0.0001**	0.01	**0.0001 (↑)**	**0.004(↑)**
Alpha-aminoadipic acid	**<0.0001**	**<0.0001 (↑)**	**<0.0001 (↑)**	**0.004 (↑)**
Asparagine	**<0.0001**	0.007	0.007	**0.0001 (↓)**
3-OHbutyrylcarnitine^a^	**0.001**	0.3	0.59	**0.0002 (↑)**
Cystine	**<0.0001**	**<0.0001 (↑)**	**0.001 (↑)**	**<0.0001 (↑)**
Hexadecanoylcarnitine	0.005	0.02	0.02	0.01
Isoleucine	**<0.0001**	**<0.0001 (↑)**	**<0.0001 (↑)**	**<0.0001 (↑)**
Leucine	**<0.0001** (↑)	**<0.0001 (↑)**	**<0.0001 (↑)**	**<0.0001 (↑)**
Lysine	**0.0006**	0.009	**0.001 (↑)**	0.008
Malonylcarnitine	**0.001**	**0.0003 (↓)**	0.008	0.37
Phenylalanine	**<0.0001**	**<0.0001 (↑)**	**<0.0001 (↑)**	**<0.0001 (↑)**
Propionylcarnitine	**0.0005**	**0.0003 (↑)**	**0.0006 (↑)**	0.03
Tyrosine	**<0.0001**	**<0.0001 (↑)**	**<0.0001 (↑)**	0.03
Valine	**<0.0001**	**0.001 (↑)**	**<0.0001 (↑)**	**<0.0001 (↑)**

**Notes.**

a 3-OHbutyrylcarnitine was specifically quantified in 58 subjects (LMW = 14, OBMW = 20, OBMUW = 11, OBDM = 13).

The species quantified in the other subjects could be either 3-OHisobutyrylcarnitine or 3-OHbutyrylcarnitine.

**Table 3:** *P*-values reflecting overall group differences, the obesity effect, metabolic unwellness trend, and diabetic effect. *P*-values < 0.005 were considered statistically significant and are bolded. Trend tests identified increasing (↑) or decreasing (↓) trends from LMW to OBMW to OBMUW groups. Group comparisons identified increasing (↑) or decreasing (↓) if the second listed group had higher or lower values respectively. For example, Diabetic Effect for glutamine and glutamate 0.0006 (↓) means that OBDM subjects have lower levels than LMW, OBMW & OBMUW subjects.

Sum or Ratio	Overall	Obesity effect (LMW vs. OBMW, OBMUW)	Metabolic Wellness Trend (Nondiabetic) (LMW → OBMW → OBMUW)	Diabetic effect (LMW, OBMW, OBMUW vs. OBDM)	Ratio meaning
GLU + GLN	**0.002**	0.08	0.08	**0.0006 (↓)**	–
HIS + ILE + LEU + LYS + MET + PHE + THR + TRP + VAL	**<0.0001**	**0.004(↑)**	**0.0002 (↑)**	**0.001(↑)**	Essential AAs
LEU +LYS	**<0.0001**	**0.0007 (↑)**	**0.0002 (↑)**	**0.0002 (↑)**	Ketogenic AAs
$ \frac{\mathrm{TRP}}{(\mathrm{TY R}+\mathrm{PHE}+\mathrm{LEU}+\mathrm{ILE}+\mathrm{V AL})} $	**<0.0001**	0.008	**0.001 (↓)**	**<0.0001 (↓)**	Determines brain serotonin synthesis (Caballero, Finer & Wurtman, 1988)
$ \frac{(\mathrm{V AL}+\mathrm{ILE}+\mathrm{LEU})}{(\mathrm{TY R}+\mathrm{PHE})} $	**0.0006**	0.5	0.09	**0.0001 (↑)**	Fischer ratio (Soeters & Fischer, 1976)
$ \frac{(\mathrm{TAU}+\mathrm{CIT}+\mathrm{LY S})}{ \left( \mathrm{ASP}+\mathrm{ILE} \right) } + \frac{(\mathrm{THR}+\mathrm{TY R}+\mathrm{HIS})}{\mathrm{GLU}} $	**<0.0001**	0.01	0.01	**<0.0001 (↓)**	Diabetes (Noguchi et al., 2006)
$ \frac{(\mathrm{C}3+\mathrm{C}5)}{\text{Total carnitine}} $	**<0.0001**	**0.0004(↑)**	**0.004 (↑)**	**0.0007(↑)**	BCAA metabolism

**Notes.**

Abbreviations ASP asparagine CIT citrulline GLU glutamate GLN glutamine HIS histidine ILE isoleucine LEU leucine LYS lysine MET methionine PHE phenylalanine TAU taurine THR threonine TRP tryptophan TYR tyrosine VAL valine C3 propionylcarnitine C5 isovalerylcarnitine

The metabolic relevance of increased levels of BCAAs in diabetes and obesity has been extensively investigated. Genetic and biochemical evidence exists to support elevated BCAA levels both as a cause and consequence of insulin resistance. BCAAs induce chronic phosphorylation of mTOR, JNK, and IRS-1, driving insulin resistance (Newgard et al., 2009). A recent genetic analysis revealed an association between a genetic variant near the *PPM1K* gene, an activator of BCAA ketoacid dehydrogenase (BCKD), and T2D, implicating BCAAs with a causal role in the development of insulin resistance (Lotta et al., 2016). Other studies have linked impaired BCAA catabolism to insulin resistance or increased adiposity through reduced expression of BCKD and branched-chain aminotransferase, another complex that catabolizes BCAAs (She et al., 2007; Pietiläinen et al., 2008). Accelerated protein breakdown, a consequence of insulin resistance, may also explain in part the increased levels of BCAAs in persons with diabetes or obesity (Lotta et al., 2016). Finally, insulin facilitates tissue uptake of BCAAs, especially in muscle, raising the possibility that insulin resistance may also be a contributing factor to the increased levels of plasma BCAAs in obese, metabolically unwell, and diabetic states (Fukagawa et al., 1986). Our data confirm involvement of the BCAAs in the pathogenesis of both obesity and diabetes, and as an important defining aspect of metabolic unwellness.

The increase in plasma BCAAs in metabolically unwell and diabetic persons has both research and clinical implications. For example, levels of essential amino acids (BCAAs, histidine, lysine, methionine, phenylalanine, threonine, tryptophan) have been used to evaluate the pathobiology of diverse conditions such as heart failure (Cheng et al., 2015). In another study, Laferrère et al. (2011) compared obese diabetics who lost an equivalent amount of weight via surgical or non-surgical means and found that BCAAs remained increased only in the non-surgical group while cholesterol and fasting glucose levels did not differ, suggesting that plasma BCAAs may serve as a more sensitive measure than traditional criteria used to assess metabolic wellness. These investigations illustrate the usefulness of BCAAs in exploring pathophysiology. However, our results regarding BCAAs and those of some others demonstrate that the BCAAs are abnormal in a variety of common pathological circumstances such as obesity, MS, and T2D. Consequently, research studies and clinical evaluations that incorporate BCAA analysis, indirectly or directly, must take into account the common clinical contexts of obesity and metabolic unwellness or risk misinterpreting pathophysiological associations.

### Lysine is elevated in metabolic unwellness while its metabolite, alpha-aminoadipic acid, is elevated in obesity, metabolic unwellness, and T2D

Lysine is an essential amino acid, while alpha-aminoadipic acid (AAD) is a product of lysine degradation. Therefore, AAD plasma levels are attributable to the catabolism of circulating or protein-derived lysine. Our data revealed that lysine increased with metabolic unwellness (*p* = 0.0006), a previously unreported finding, and had nearly significant increases in obese and diabetic states (*p* = 0.009 and *p* = 0.008, respectively) (Table 2, Fig. 1). Plasma lysine levels are not noted to be abnormal in most studies of either obesity, T2D or MS (Tables S1, S2); a single study of obese African American women with T2D reported moderately decreased lysine but did not address its possible significance (Fiehn et al., 2010).

We noted that plasma AAD was significantly increased in all three states, with *p* < 0.0001 for obesity and metabolic unwellness trend, and *p* = 0.004 for the T2D effect, respectively (Table 2). Only one other study has reported similar results. Wang et al. (2013) observed that AAD predicted the development of diabetes in normoglycemic individuals and hypothesized that AAD levels increase in response to hyperglycemia, increasing insulin secretion and contributing to a compensatory mechanism to maintain glucose homeostasis in early insulin resistance. One explanation for the elevation in plasma lysine is its mobilization to provide AAD to promote insulin secretion in early insulin resistance, with perhaps less of a role in the setting of advanced insulin resistance in T2D. This hypothesis is consistent with our observation of a more modest difference in AAD levels between diabetic and non-diabetics compared to lean and obese subjects.

It is important to note that the pathways of BCAA and of lysine/AAD metabolism are distinct. This, in turn, indicates that disparate biochemical pathways are dysregulated in states of obesity, metabolic unwellness, and diabetes.

### Changes in phenylalanine and tyrosine levels with obesity, metabolic unwellness, and T2D reflect the pathophysiology of these processes and affect ratios implicated with hepatic function and serotonin synthesis

Phenylalanine, an essential amino acid, and tyrosine, a hydroxylation product of phenylalanine metabolism, are largely metabolized in the liver, as is the other aromatic amino acid (AAA) tryptophan. We noted the plasma phenylalanine level to be increased in obese, metabolically unwell, and diabetic states (*p* < 0.0001), while tyrosine was increased in obese and metabolically unwell states (*p* < 0.0001) but did not significantly differ between diabetics and non-diabetics (*p* = 0.03) (Table 2, Fig. 1). Increased plasma levels of phenylalanine and tyrosine have been observed in most analyses of amino acid biomarkers in obesity and T2D (Table S1).

The basis for the findings that we and others have noted is incompletely understood. Changes in these amino acids could be explained by several factors. One hypothesis is that the increased circulating levels of the BCAAs compete with the aromatic amino acids for uptake into tissues through the shared large neutral amino acid transporter (LAT1) (Fernstrom, 2005; Newgard et al., 2009). Another possible explanation is that increasing liver dysfunction associated with metabolic unwellness results in decreased phenylalanine and tyrosine metabolism, leading to their elevated levels in plasma.

Still other factors may be operative and explain the increased levels of tyrosine and, possibly, phenylalanine. We noted, for example, that plasma cystine is significantly associated with obesity (*p* < 0.0001), metabolic unwellness (*p* = 0.001), and diabetes (*p* < 0.0001) (Table 2, Fig. 1). Cystine levels could be elevated as a result of increased oxidative stress, or very early renal dysfunction (Buckley & Milligan, 1978; Hargrove & Wichman, 1987; Wijekoon, Brosnan & Brosnan, 2007; Pastore et al., 2015). Cysteine inhibits tyrosine aminotransferase activity, and can lead to an increased plasma tyrosine level (Buckley & Milligan, 1978; Hargrove & Wichman, 1987). No satisfying explanation for the elevation of tyrosine with obesity and metabolic unwellness but not diabetes is apparent. This suggests that obese, metabolically unwell, and T2D subjects have metabolic signatures that evolve in a manner that is not entirely straightforward. Taken together, these findings indicate that metabolic unwellness, through insulin resistance, the generation of reactive oxygen species, or early end-organ dysfunction, leads to an elevation in phenylalanine and tyrosine.

Changes in the levels of the aromatic amino acids in the three states that we investigated have both research and clinical relevance. Plasma AAA levels are increased in persons with hepatic cirrhosis and, as such, the Fischer ratio (BCAAs/AAAs) is known to decrease with worsening liver dysfunction and is used to assess disease severity (Soeters & Fischer, 1976; Dejong et al., 2007). In this study, both AAAs and BCAAs increased in obesity, metabolic unwellness, and T2D, but the Fischer ratio only changed significantly in the diabetic state (*p* = 0.0001) (Table 3). A possible explanation for this finding is that only in a state of extreme insulin resistance will the elevation of BCAAs so markedly surpass that of the AAAs. From a practical perspective, this finding advises caution in clinically using this ratio to evaluate hepatic dysfunction in diabetics.

In addition, serotonin synthesis in the brain depends on the tryptophan pool which, in turn, is determined by competition between tryptophan and other large neutral amino acids (LNAAs; tryptophan, tyrosine, phenylalanine, and the BCAAs) at the blood–brain barrier (Pardridge & Oldendorf, 1975; Fernstrom & Wurtman, 1997). Therefore, the tryptophan/LNAA ratio has been utilized as a proxy to visualize changes in the brain tryptophan pool (Ashley et al., 1985; Caballero, Finer & Wurtman, 1988; Breum et al., 2003). We found this ratio to be significantly decreased with metabolic unwellness (*p* = 0.001) and T2D (*p* < 0.0001), and nearly significant with obesity (*p* = 0.008) (Table 3). The tryptophan/LNAA ratio has not been evaluated in recent studies, but several older studies are consistent with our findings, though there are no data regarding the relevance of this ratio to metabolic unwellness. This is a potentially actionable finding as tryptophan supplementation could possibly correct this ratio and provide clinical benefit. To our knowledge, no study of the effect of tryptophan supplementation on weight loss or amelioration of metabolic syndrome has been published (Caballero, Finer & Wurtman, 1988). Several monogenic metabolic disorders such as phenylketonuria are characterized by markedly abnormal plasma amino acids levels that result in toxic brain levels. While the mainstay of phenylketonuria treatment, for example, is the reduction of phenylalanine intake, recent investigations using animal models suggest that supplementation with large neutral amino acids that compete with the excessive levels of phenylalanine at the shared transport system may be an effective alternative treatment (Van Vliet et al., 2016). Although the magnitude of perturbation of brain amino acids would be expected to be less in persons with multifactorially determined obesity or metabolic syndrome than in classic monogenic metabolic disorders, a similar principle could apply to treating these patients and further work in this area appears warranted based on our findings.

In sum, these findings indicate that metabolic unwellness, through various possible mechanisms, leads to an elevation in phenylalanine and tyrosine. In turn, the elevation of these species may alter the interpretation of the Fischer ratio, an indicator of hepatic function, in diabetics. Moreover, the decreased tryptophan/LNAA ratio in metabolically unwell subjects indicates a possible decrease in serotonin synthesis, which may be clinically actionable.

### Malonylcarnitine is decreased in obesity while 3-OHbutyrylcarnitine increased in T2D

Malonyl-CoA, the precursor of malonylcarnitine, is formed from acetyl-CoA by acetyl-CoA carboxylase and inhibits carnitine palmitoyltransferase I which, in turn, catalyzes the rate-limiting step of mitochondrial fatty acid beta-oxidation (Saggerson, 2008). We found that malonylcarnitine is increased in lean vs. obese subjects (*p* = 0.0003); its levels suggested but did not reach significance in terms of trending with metabolic wellness (*p* = 0.008) (Table 2, Fig. 1). 3-OHbutyrylcarnitine is increased in ketosis as it correlates with 3-hydroxybutyrate levels (Hack et al., 2006). We found that 3-OHbutyrylcarnitine was elevated in T2D (*p* = 0.0002) (Table 2, Fig. 1). Few studies have reported changes in these metabolites. One study noted elevated levels of malonylcarnitine in incident T2D (Sun et al., 2016) and Mai et al. (2013) reported that the sum of malonylcarnitine and 3-OHbutyrylcarnitine increased in T2D subjects compared to subjects with normal glucose tolerance, impaired fasting glucose, and impaired glucose tolerance.

The finding of increased 3-OHbutyrylcarnitine in T2D is not unexpected and is consistent with increased ketones sometimes noted in this population (Mahendran et al., 2013). That malonylcarnitine is increased in lean normal vs. obese subjects is, to our knowledge, previously unreported and unexpected. A possible explanation may be that by inhibiting carnitine palmitoyltransferase I, it serves to shunt fatty acids away from oxidative metabolism and promote an anabolic utilization of these compounds in the well, non-diabetic state.

### Homocysteine levels did not significantly differ with obesity, metabolic wellness, or T2D

In this study, the median levels of plasma homocysteine did not significantly change with obesity or T2D, and did not trend with metabolic unwellness (*p* = 0.26 overall, Table S5A). For decades, homocysteine has been of interest because an elevated serum level is considered a risk factor for cardiovascular disease (Boushey et al., 1995; Homocysteine Studies Collaboration, 2002; Wald, Law & Morris, 2002). This has been related to its role in causing endothelial damage (Félétou & Vanhoutte, 2006). Studies of the association of serum homocysteine levels with insulin resistance have had conflicting results (Bar-On et al., 2000; Godsland et al., 2001; Meigs et al., 2001; Sanchez-Margalet et al., 2002). Mechanistically, elevated serum homocysteine could be a cause or consequence of insulin resistance. Studies in both rats and cultured human hepatocytes show that hyperinsulinemia affects the activity of 5,10-methlenetetrahydrofolate reductase (MTHFR) and cystathionine-beta-synthase (CBS), two key enzymes in homocysteine metabolism (Fonseca et al., 2000; Dicker-Brown et al., 2001). Additionally, one case-control study showed that the MTHFR C677T gene polymorphism, which results in increased serum homocysteine levels, is associated with metabolic syndrome (Yang et al., 2014). There has been much interest in researching whether lowering serum homocysteine levels has a beneficial effect on cardiovascular disease. One recent meta-analysis of randomized-controlled trials studying the effect of B12, B9, and/or B6 supplementation, which lowers blood homocysteine, found no evidence that supplementation prevents myocardial infarction or lowers mortality rates in those living with cardiovascular disease, while antihypertensives combined with a homocysteine-lowering regimen may reduce stroke (Martí-Carvajal et al., 2017).

### Strengths and limitations of this work

We recognize several limitations to our study. First, although our subject groups included both ends of the spectrum of metabolic wellness, more granular analysis by inclusion of several additional “intermediate” groups such as individuals who are lean and metabolically unwell, individuals who are overweight (but not obese) and metabolically well, and individuals who are overweight and unwell may have added to our study. Related to this, the fact that we lacked waist circumference data and had subjects of different races in our sample groups means that there is a possibility that our groups had slightly different metabolomes than their corresponding categories in the general population. Additionally, while we applied rigorous statistical methodology, it is possible that the size of our groups did not provide adequate statistical power to detect some very modest but still clinically relevant metabolic changes or changes that occur in subpopulations.

Second, this study, like nearly all investigations in this area, analyzed fasting samples. Recent work indicates that physiological challenges such as glucose or lipid loads, exercise or temperature stress can reveal increased inter-individual metabolic variation, even in phenotypically similar subjects (Krug et al., 2012). This variation may be of clinical relevance but cannot be captured by exclusively analyzing fasting samples.

Third, while we analyzed a large number of metabolites, the targeted nature of the metabolomics analysis necessarily precludes discovery of analytes that may be of significance that were not analyzed. One recent cohort study which applied an unbiased systems approach and analyzed 542 metabolites uncovered novel biomarkers including species such as *α*-tocopherol, bradykinin and others, illustrating the usefulness of this approach (Peddinti et al., 2017). Fourth, the statistically significant metabolic associations that we noted do not establish causality of those associations. Although we see the changes in metabolite levels between populations differing in severity in terms of metabolic wellness and T2D (Table 2), this study does not enable one to make conclusions about the contributory nature of metabolic changes in the progression of these conditions. This limitation is present in nearly all biomarker studies of similar design.

This study has several strengths. Unlike prior metabolomic analyses of obesity or metabolic syndrome, this study incorporates an inclusion of well-defined populations of individuals along the pathophysiological spectrum that current definitions of metabolic wellness are designed to characterize: from lean, metabolically well individuals to those that have developed T2D. Furthermore, we used one of the widely-used criteria of metabolic wellness to categorize our subjects, standardizing our results with other studies of the pathophysiology of metabolic syndrome. Additionally, we evaluated a larger number of amino acid and acylcarnitine species than is measured in many similar biomarker studies and, unlike most studies, also analyzed multiple clinically relevant ratios of these analytes. These measurements allowed us to uncover findings that have not been noted in previous work. Lastly, use of a low a priori significance level, 0.005, reduced the likelihood of false discovery.

## Conclusions

By group comparison and trend analysis of plasma amino acids and acylcarnitine levels in prespecified groups along a progression from LMW to OBDM, we have both supported findings in the literature and uncovered novel results. There is a “dose-dependent relationship” between many metabolite levels and ratios with increasing metabolic unwellness, culminating in T2D, although an unsupervised cluster analysis did not separate the subjects into the four prespecified groups (Table S4). In terms of specific analytes, we found that the BCAAs, which have been described as both a cause and consequence of insulin resistance, are elevated in obesity, metabolic unwellness, and T2D. Our findings regarding lysine and its metabolite alpha-aminoadipic acid support the hypothesis that alpha-aminoadipic acid is involved in the pathophysiology of these conditions and may act in early insulin resistance. Tyrosine was elevated in obesity and metabolic unwellness, earlier in MS progression than its precursor, phenylalanine, which was also elevated in T2D. These findings could be attributed to early organ dysfunction or more complex changes in biochemical pathways induced by insulin resistance. Malonylcarnitine decreased with obesity, a novel result warranting further investigation. Importantly, certain ratios describing physiologic functions were altered as well. The Fischer ratio of liver dysfunction was altered in T2D, indicating that it should be used with caution in evaluating T2D patients. The tryptophan/LNAA ratio, an indicator of serotonin synthesis, was decreased in obesity, MS, and T2D, and represents a potentially clinically actionable finding. Our work adds to that of others in the field in establishing the disparate biochemical pathways, not just those of BCAAs, are implicated in the pathophysiology of obesity, metabolic unwellness, and T2D.

Overall, we found: (1) even obese, metabolically healthy individuals by ATP III criteria had metabolite levels that trended toward those of obese, diabetic individuals, challenging the notion of the “obese, metabolically well”; (2) there appears to be a “dose-dependent relationship” between many metabolite levels and ratios with increasing metabolic unwellness, culminating in T2D; and (3) the progression from obesity to T2D is marked by complex biochemical changes that are not always explained by the straightforward worsening of metabolic regulation in the face of increasing insulin resistance. This is shown by the elevation of tyrosine with obesity and metabolic unwellness and the decrease in malonylcarnitine. This suggests the utility of measuring metabolite parameters over time in subjects when studying the metabolic effects of interventions for obesity, metabolic syndrome, or T2D and may provide a more complete view of an individual’s metabolic wellness, especially if incorporated with other data such as HDL levels and measures of insulin resistance.

##  Supplemental Information

10.7717/peerj.5410/supp-1 Table S1 Fasting plasma/serum amino acid and select acylcarnitine findings in persons with obesity, incident T2DM and T2DM

10.7717/peerj.5410/supp-2 Table S2 Amino acid contributions to metabolically unhealthy phenotypes Only studies reporting data on the contributions of individual plasma/serum amino acids to metabolic unwellness are included in this analysis, resulting in the exclusion of several important studies (Batch et al., 2013; Allam-Ndoul et al., 2016).

10.7717/peerj.5410/supp-3 Table S3A Mean sample storage time at 4 °C by group measured in days

10.7717/peerj.5410/supp-4 Table S3B Sensitivity analysis of samples stored less than 3 days at 4 °C: Amino acids and acylcarnitines Numbers of samples by group: LMW (21), OBMW (16), OBMUW (11), OBDM (6). *P*-values for overall significance (Kruskal-Wallis), Nondiabetic trend (JT trend test), diabetics vs. nondiabetics (Wilcoxon Rank-Sum), and LMW vs. obese nondiabetic (OBNDM, consists of OBMW and OBMUW) subjects (Wilcoxon Rank-Sum). *P*-values <0.005 were considered significant and are bolded. Statistical tests were conducted as described in Methods ‘Statistical analyses’.

10.7717/peerj.5410/supp-5 Table S3C Sensitivity analysis of samples stored less than 3 days at 4 °C: sums and ratios Numbers of samples by group: LMW (21), OBMW (16), OBMUW (11), OBDM (6). *P*-values for overall significance (Kruskal-Wallis), Nondiabetic trend (JT trend test), diabetics vs. nondiabetics (Wilcoxon Rank-Sum), and LMW vs. obese nondiabetic (OBNDM, consists of OBMW and OBMUW) subjects (Wilcoxon Rank-Sum). *P*-values <0.005 were considered significant and are bolded. Statistical tests were conducted as described in Table 2. Abbreviations are as follows: GLU, glutamate; GLN, glutamine; HIS, histidine; ILE, isoleucine; LEU, leucine; LYS, lysine; MET, methionine; PHE, phenylalanine; THR, threonine; TRP, tryptophan; TYR, tyrosine; VAL, valine; C3, propionylcarnitine; C5, isovalerylcarnitine.

10.7717/peerj.5410/supp-6 Table S4 K-means cluster analysis K-means cluster analysis of the quantitative values of each metabolite does not separate our data into groups based on metabolic wellness by ATP III criteria. All analytes are included except for homocysteine and 3-hydroxybutyrate, which have incomplete data. Each value in the group columns (LMW, OBMW, OBMUW and OBDM) is presented as a count unless otherwise specified.

10.7717/peerj.5410/supp-7 Table S5A Median (lower quartile, upper quartile) levels of plasma amino acids and related compounds All amino acids and related compounds were measured in all of the 90 samples except for homocysteine which was measured in all but 5 samples due to lack of volume, yielding the following sample sizes (*N* = 85), LMW = 24, OBMW = 24, OBMUW = 18, OBDM = 19. Amino acid levels are reported as micromoles/liter and are rounded to 0.1. Values are given as median (Q_1_, Q_3_) Bolded values were significantly different upon Kruskal-Wallis testing (*p* < 0.005).

10.7717/peerj.5410/supp-8 Table S5B Median (lower quartile, upper quartile) levels of acylcarnitine species Values are given as median (Q_1_, Q_3_). Bolded values were significantly different upon Kruskal-Wallis testing (*p* < 0.005). Acylcarnitine levels are reported as nanomoles/liter, except for free carnitine and acetylcarnitine which are reported as micromoles/liter. Values are rounded to 0.1.

10.7717/peerj.5410/supp-9 Table S5C Median sums or ratios Acylcarnitine levels are reported as nanomoles/liter, except for C0 and C2 that are reported as micromoles/liter. Values are rounded to 0.1 and are given as median (Q1, Q3) Bolded values were significantly different upon Kruskal-Wallis testing (*p* < 0.005). Both glucogenic and ketogenic amino acids are isoleucine, phenylalanine, threonine, tryptophan, and tyrosine. Glucogenic amino acids are alanine, arginine, asparagine, aspartic acid, cystine, glutamate, glutamine, glycine, histidine, methionine, proline, serine, and valine. Abbreviations are as follows: ABU, aminobutyrate; ALA, alanine; ASP, asparagine; CIT, citrulline; GLU, glutamate; GLN, glutamine; HIS, histidine; ILE, isoleucine; LEU, leucine; LYS, lysine; MET, methionine; ORN, ornithine; PHE, phenylalanine; TAU, taurine; THR, threonine; TRP, tryptophan; TYR, tyrosine VAL, valine; C2, acetylcarnitine; C3, propionylcarnitine C4, iso/butyrylcarnitine; C5, isovalerylcarnitine, C6, hexanoylcarnitine; C8, octanoylcarnitine; C10, decanoylcarnitine; C10:1, decenoylcarnitine; C16, hexadecanoylcarnitine (palmitoylcarnitine), C16-OH=3-OH-hexadecenoylcarnitine.

10.7717/peerj.5410/supp-10 Data S1 Raw data

## Additional Information and Declarations

### Data Availability

The following information was supplied regarding data availability: The raw data are provided in a Supplemental File.

## References

[ref-1] Adams SH, Hoppel CL, Lok KH, Zhao L, Wong SW, Minkler PE, Hwang DH, Newman JW, Garvey WT (2009). Plasma acylcarnitine profiles suggest incomplete long-chain fatty acid beta-oxidation and altered tricarboxylic acid cycle activity in type 2 diabetic African-American women. The Journal of Nutrition.

[ref-2] Alberti KG, Zimmet PZ (1998). Definition, diagnosis and classification of diabetes mellitus and its complications. Part 1: diagnosis and classification of diabetes mellitus provisional report of a WHO consultation. Diabetic Medicine.

[ref-3] Ashley DV, Fleury MO, Golay A, Maeder E, Leathwood PD (1985). Evidence for diminished brain 5-hydroxytryptamine biosynthesis in obese diabetic and non-diabetic humans. The American Journal of Clinical Nutrition.

[ref-4] Bailey K (1994). Numerical taxonomy ad cluster analysis. Typologies and tax-onomies: an introduction to classification.

[ref-5] Balkau B, Charles MA (1999). Comment on the provisional report from the WHO consultation. European Group for the Study of Insulin Resistance (EGIR). Diabetic Medicine.

[ref-6] Bar-On H, Kidron M, Friedlander Y, Ben-Yehuda A, Selhub J, Rosenberg IH, Friedman G (2000). Plasma total homocysteine levels in subjects with hyperinsulinemia. Journal of Internal Medicine.

[ref-7] Boushey CJ, Beresford SA, Omenn GS, Motulsky AG (1995). A quantitative assessment of plasma homocysteine as a risk factor for vascular disease. Probable benefits of increasing folic acid intakes. Journal of the American Medical Association.

[ref-8] Breum L, Rasmussen MH, Hilsted J, Fernstrom JD (2003). Twenty-four-hour plasma tryptophan concentrations and ratios are below normal in obese subjects and are not normalized by substantial weight reduction. The American Journal of Clinical Nutrition.

[ref-9] Buckley WT, Milligan LP (1978). Participation of cysteine and cystine in inactivation of tyrosine aminotransferase in rat liver homogenates. The Biochemical Journal.

[ref-10] Caballero B, Finer N, Wurtman RJ (1988). Plasma amino acids and insulin levels in obesity: response to carbohydrate intake and tryptophan supplements. Metabolism: Clinical and Experimental.

[ref-11] Cheng M-L, Wang C-H, Shiao M-S, Liu M-H, Huang Y-Y, Huang C-Y, Mao C-T, Lin J-F, Ho H-Y, Yang N-I (2015). Metabolic disturbances identified in plasma are associated with outcomes in patients with heart failure: diagnostic and prognostic value of metabolomics. Journal of the American College of Cardiology.

[ref-12] Dejong CHC, Van de Poll MCG, Soeters PB, Jalan R, Olde Damink SWM (2007). Aromatic amino acid metabolism during liver failure. The Journal of Nutrition.

[ref-13] Dicker-Brown A, Fonseca VA, Fink LM, Kern PA (2001). The effect of glucose and insulin on the activity of methylene tetrahydrofolate reductase and cystathionine- *β*-synthase: studies in hepatocytes. Atherosclerosis.

[ref-14] Fan J, Song Y, Chen Y, Hui R, Zhang W (2013). Combined effect of obesity and cardio-metabolic abnormality on the risk of cardiovascular disease: a meta-analysis of prospective cohort studies. International Journal of Cardiology.

[ref-15] Félétou M, Vanhoutte PM (2006). Endothelial dysfunction: a multifaceted disorder. American Journal of Physiology.

[ref-16] Felig P, Marliss E, Cahill GFJ (1969). Plasma amino acid levels and insulin secretion in obesity. New England Journal of Medicine.

[ref-17] Fernstrom JD (2005). Branched-chain amino acids and brain function. The Journal of Nutrition.

[ref-18] Fernstrom JD, Wurtman RJ (1997). Brain serotonin content: physiological regulation by plasma neutral amino acids. 1971. Obesity Research.

[ref-19] Fiehn O, Garvey WT, Newman JW, Lok KH, Hoppel CL, Adams SH (2010). Plasma metabolomic profiles reflective of glucose homeostasis in non-diabetic and type 2 diabetic obese African-American women. PLOS ONE.

[ref-20] Fonseca V, Dicker-Brown A, Ranganathan S, Song W, Barnard RJ, Fink L, Kern PA (2000). Effects of a high-fat-sucrose diet on enzymes in homocysteine metabolism in the rat. Metabolism: Clinical and Experimental.

[ref-21] Fukagawa NK, Minaker KL, Young VR, Rowe JW (1986). Insulin dose-dependent reductions in plasma amino acids in man. The American Journal of Physiology.

[ref-22] The GBD 2015 Obesity Collaborators (2017). Health effects of overweight and obesity in 195 countries over 25 years. New England Journal of Medicine.

[ref-23] Godsland IF, Rosankiewicz JR, Proudler AJ, Johnston DG (2001). Plasma total homocysteine concentrations are unrelated to insulin sensitivity and components of the metabolic syndrome in healthy men. The Journal of Clinical Endocrinology & Metabolism.

[ref-24] Grundy SM, Cleeman JI, Daniels SR, Donato KA, Eckel RH, Franklin BA, Gordon DJ, Krauss RM, Savage PJ, Smith SC, Spertus JA, Costa F (2005). Diagnosis and management of the metabolic syndrome. Circulation.

[ref-25] Hack A, Busch V, Pascher B, Busch R, Bieger I, Gempel K, Baumeister FAM (2006). Monitoring of ketogenic diet for carnitine metabolites by subcutaneous microdialysis. Pediatric Research.

[ref-26] Hargrove JL, Wichman RD (1987). A cystine-dependent inactivator of tyrosine aminotransferase co-purifies with gamma-cystathionase (cystine desulfurase). The Journal of Biological Chemistry.

[ref-27] Homocysteine Studies Collaboration (2002). Homocysteine and risk of ischemic heart disease and stroke: a meta-analysis. Journal of the American Medical Association.

[ref-28] Jain AK (2010). Data clustering: 50 years beyond K-means. Pattern Recognition Letters.

[ref-29] Jeevanandam M, Ramias L, Schiller WR (1991). Altered plasma free amino acid levels in obese traumatized man. Metabolism: Clinical and Experimental.

[ref-30] Kramer CK, Zinman B, Retnakaran R (2013). Are metabolically healthy overweight and obesity benign conditions?: a systematic review and meta-analysis. Annals of Internal Medicine.

[ref-31] Krug S, Kastenmüller G, Stückler F, Rist MJ, Skurk T, Sailer M, Raffler J, Römisch-Margl W, Adamski J, Prehn C, Frank T, Engel K-H, Hofmann T, Luy B, Zimmermann R, Moritz F, Schmitt-Kopplin P, Krumsiek J, Kremer W, Huber F, Oeh U, Theis FJ, Szymczak W, Hauner H, Suhre K, Daniel H (2012). The dynamic range of the human metabolome revealed by challenges. FASEB Journal.

[ref-32] Laferrère B, Reilly D, Arias S, Swerdlow N, Gorroochurn P, Bawa B, Bose M, Teixeira J, Stevens RD, Wenner BR, Bain JR, Muehlbauer MJ, Haqq A, Lien L, Shah SH, Svetkey LP, Newgard CB (2011). Differential metabolic impact of gastric bypass surgery versus dietary intervention in obese diabetic subjects despite identical weight loss. Science Translational Medicine.

[ref-33] Lavie CJ, De Schutter A, Milani RV (2015). Healthy obese versus unhealthy lean: the obesity paradox. Nature Reviews Endocrinology.

[ref-34] Lotta LA, Scott RA, Sharp SJ, Burgess S, Luan J ’an, Tillin T, Schmidt AF, Imamura F, Stewart ID, Perry JRB, Marney L, Koulman A, Karoly ED, Forouhi NG, Sjögren RJO, Näslund E, Zierath JR, Krook A, Savage DB, Griffin JL, Chaturvedi N, Hingorani AD, Khaw K-T, Barroso I, McCarthy MI, O’Rahilly S, Wareham NJ, Langenberg C (2016). Genetic predisposition to an impaired metabolism of the branched-chain amino acids and risk of type 2 diabetes: a mendelian randomisation analysis. PLOS Medicine.

[ref-35] Mahendran Y, Vangipurapu J, Cederberg H, Stancáková A, Pihlajamäki J, Soininen P, Kangas AJ, Paananen J, Civelek M, Saleem NK, Pajukanta P, Lusis AJ, Bonnycastle LL, Morken MA, Collins FS, Mohlke KL, Boehnke M, Ala-Korpela M, Kuusisto J, Laakso M (2013). Association of ketone body levels with hyperglycemia and type 2 diabetes in 9,398 Finnish men. Diabetes.

[ref-36] Mai M, Tönjes A, Kovacs P, Stumvoll M, Fiedler GM, Leichtle AB (2013). Serum levels of acylcarnitines are altered in prediabetic conditions. PLOS ONE.

[ref-37] Martí-Carvajal AJ, Solà I, Lathyris D, Dayer M (2017). Homocysteine-lowering interventions for preventing cardiovascular events. Cochrane database of systematic reviews.

[ref-38] Meigs JB, Jacques PF, Selhub J, Singer DE, Nathan DM, Rifai N, D’Agostino RB, Wilson PW (2001). Fasting plasma homocysteine levels in the insulin resistance syndrome: the Framingham offspring study. Diabetes Care.

[ref-39] Mihalik SJ, Michaliszyn SF, De las Heras J, Bacha F, Lee S, Chace DH, DeJesus VR, Vockley J, Arslanian SA (2012). Metabolomic profiling of fatty acid and amino acid metabolism in youth with obesity and type 2 diabetes: evidence for enhanced mitochondrial oxidation. Diabetes Care.

[ref-40] Mørkedal B, Vatten LJ, Romundstad PR, Laugsand LE, Janszky I (2014). Risk of myocardial infarction and heart failure among metabolically healthy but obese individuals: HUNT (Nord-Trøndelag Health Study), Norway. Journal of the American College of Cardiology.

[ref-41] Narayan SB, Ditewig-Meyers G, Graham KS, Scott R, Bennett MJ (2011). Measurement of plasma amino acids by ultraperformance^®^ liquid chromatography. Clinical Chemistry and Laboratory Medicine.

[ref-42] Newgard CB, An J, Bain JR, Muehlbauer MJ, Stevens RD, Lien LF, Haqq AM, Shah SH, Arlotto M, Slentz CA, Rochon J, Gallup D, Ilkayeva O, Wenner BR, Yancy WS, Eisenson H, Musante G, Surwit RS, Millington DS, Butler MD, Svetkey LP (2009). A branched-chain amino acid-related metabolic signature that differentiates obese and lean humans and contributes to insulin resistance. Cell Metabolism.

[ref-43] Noguchi Y, Zhang Q-W, Sugimoto T, Furuhata Y, Sakai R, Mori M, Takahashi M, Kimura T (2006). Network analysis of plasma and tissue amino acids and the generation of an amino index for potential diagnostic use. The American Journal of Clinical Nutrition.

[ref-44] Ogden CL, Carroll MD, Flegal KM (2003). Epidemiologic trends in overweight and obesity. Endocrinology and Metabolism Clinics of North America.

[ref-45] Pardridge WM, Oldendorf WH (1975). Kinetic analysis of blood–brain barrier transport of amino acids. Biochimica Et Biophysica Acta.

[ref-46] Pastore A, Noce A, Di Giovamberardino G, De Stefano A, Callà C, Zenobi R, Dessì M, Di Daniele N (2015). Homocysteine, cysteine, folate and vitamin B_1__2_ status in type 2 diabetic patients with chronic kidney disease. Journal of Nephrology.

[ref-47] Peddinti G, Cobb J, Yengo L, Froguel P, Kravić J, Balkau B, Tuomi T, Aittokallio T, Groop L (2017). Early metabolic markers identify potential targets for the prevention of type 2 diabetes. Diabetologia.

[ref-48] Pietiläinen KH, Naukkarinen J, Rissanen A, Saharinen J, Ellonen P, Keränen H, Suomalainen A, Götz A, Suortti T, Yki-Järvinen H, Orešič M, Kaprio J, Peltonen L (2008). Global transcript profiles of fat in monozygotic twins discordant for BMI: pathways behind acquired obesity. PLOS Medicine.

[ref-49] Rauschert S, Uhl O, Koletzko B, Hellmuth C (2014). Metabolomic biomarkers for obesity in humans: a short review. Annals of Nutrition & Metabolism.

[ref-50] Saggerson D (2008). Malonyl-CoA, a key signaling molecule in mammalian cells. Annual Review of Nutrition.

[ref-51] Sanchez-Margalet V, Valle M, Ruz FJ, Gascon F, Mateo J, Goberna R (2002). Elevated plasma total homocysteine levels in hyperinsulinemic obese subjects. The Journal of Nutritional Biochemistry.

[ref-52] Schooneman MG, Achterkamp N, Argmann CA, Soeters MR, Houten SM (2014). Plasma acylcarnitines inadequately reflect tissue acylcarnitine metabolism. Biochimica Et Biophysica Acta.

[ref-53] Scott D, Heese B, Garg U (2016). Quantification of free carnitine and acylcarnitines in plasma or serum using HPLC/MS/MS. Methods in Molecular Biology.

[ref-54] She P, Van Horn C, Reid T, Hutson SM, Cooney RN, Lynch CJ (2007). Obesity-related elevations in plasma leucine are associated with alterations in enzymes involved in branched-chain amino acid metabolism. American Journal of Physiology.

[ref-55] Soeters PB, Fischer JE (1976). Insulin, glucagon, aminoacid imbalance, and hepatic encephalopathy. Lancet.

[ref-56] Suhre K, Meisinger C, Döring A, Altmaier E, Belcredi P, Gieger C, Chang D, Milburn MV, Gall WE, Weinberger KM, Mewes H-W, Hrabé de Angelis M, Wichmann H-E, Kronenberg F, Adamski J, Illig T (2010). Metabolic footprint of diabetes: a multiplatform metabolomics study in an epidemiological setting. PLOS ONE.

[ref-57] Sun L, Liang L, Gao X, Zhang H, Yao P, Hu Y, Ma Y, Wang F, Jin Q, Li H, Li R, Liu Y, Hu FB, Zeng R, Lin X, Wu J (2016). Early prediction of developing type 2 diabetes by plasma acylcarnitines: a population-based study. Diabetes Care.

[ref-58] Tewari PC, Zhang B, Bluestein BI (2004). Analytical and clinical evaluation of the Bayer ADVIA Centaur homocysteine assay. Clinica Chimica Acta.

[ref-59] Van Vliet D, Bruinenberg VM, Mazzola PN, Van Faassen MH, De Blaauw P, Pascucci T, Puglisi-Allegra S, Kema IP, Heiner-Fokkema MR, Van der Zee EA, Van Spronsen FJ (2016). Therapeutic brain modulation with targeted large neutral amino acid supplements in the Pah-enu2 phenylketonuria mouse model. The American Journal of Clinical Nutrition.

[ref-60] Wald DS, Law M, Morris JK (2002). Homocysteine and cardiovascular disease: evidence on causality from a meta-analysis. BMJ.

[ref-61] Wang TJ, Ngo D, Psychogios N, Dejam A, Larson MG, Vasan RS, Ghorbani A, O’Sullivan J, Cheng S, Rhee EP, Sinha S, McCabe E, Fox CS, O’Donnell CJ, Ho JE, Florez JC, Magnusson M, Pierce KA, Souza AL, Yu Y, Carter C, Light PE, Melander O, Clish CB, Gerszten RE (2013). 2-Aminoadipic acid is a biomarker for diabetes risk. The Journal of Clinical Investigation.

[ref-62] Wijekoon EP, Brosnan ME, Brosnan JT (2007). Homocysteine metabolism in diabetes. Biochemical Society Transactions.

[ref-63] Xu F, Tavintharan S, Sum CF, Woon K, Lim SC, Ong CN (2013). Metabolic signature shift in type 2 diabetes mellitus revealed by mass spectrometry-based metabolomics. The Journal of Clinical Endocrinology and Metabolism.

[ref-64] Yang B, Fan S, Zhi X, Wang D, Li Y, Wang Y, Wang Y, Wei J, Zheng Q, Sun G (2014). Associations of MTHFR C677T and MTRR A66G gene polymorphisms with metabolic syndrome: a case-control study in northern China. International Journal of Molecular Sciences.

[ref-65] Zhong F, Xu M, Bruno RS, Ballard KD, Zhu J (2017). Targeted high performance liquid chromatography tandem mass spectrometry-based metabolomics differentiates metabolic syndrome from obesity. Experimental Biology and Medicine.

[ref-66] Zimmet P, Magliano D, Matsuzawa Y, Alberti G, Shaw J (2005). The metabolic syndrome: a global public health problem and a new definition. Journal of Atherosclerosis and Thrombosis.

