# Supplementary material for: Metabolomic analysis of obesity, metabolic syndrome, and type 2 diabetes: amino acid and acylcarnitine levels change along a spectrum of metabolic wellness
Source: PeerJ. 2018 Aug 31;6:e5410. doi: 10.7717/peerj.5410 (PMC6120443; doi:10.7717/peerj.5410)
Supplement: Table S1 [file peerj-06-5410-s001.docx]

| **Species** | **Finding** | **Authors, Year** |
| --- | --- | --- |
| Alanine | Inc in obese adults | (Newgard et al., 2009) |
|  | Inc. in T2D adults | (Wang-Sattler et al., 2012)  (Xu et al., 2013)  (Villarreal-Pérez et al., 2014) |
|  | Inc. incident T2D adults | (Palmer et al., 2015)  (Qiu et al., 2016) |
|  | Inc. in incident T2D European and S. Asian adult males | (Tillin et al., 2015) |
| Alpha-aminoadipate | Inc. in incident T2D adults | (Wang et al., 2013) |
| Aspartate or Asparagine | Inc. in obese adults | (Newgard et al., 2009) |
|  | Dec. in incident T2D adults | (Palmer et al., 2015) |
| Cysteine | Dec. in T2D adults | (Suhre et al., 2010) |
| Cystine | Dec. in obese adults | (Jeevanandam, Ramias & Schiller, 1991) |
|  | Inc. in obese, T2D adult females | (Fiehn et al., 2010) |
| Glutamate or Glutamine | Inc in obese adults | (Newgard et al., 2009) |
|  | Inc. in incident T2D adults | (Palmer et al., 2015) |
| Glycine | Dec. in obese adults | (Felig, Marliss & Cahill, 1969)  (Newgard et al., 2009) |
|  | Dec. in T2D adults | (Wang-Sattler et al., 2012) |
|  | Dec. in obese T2D adult females | (Fiehn et al., 2010) |
|  | Dec. in incident T2D | (Wang-Sattler et al., 2012)  (Floegel et al., 2014)  (Palmer et al., 2015)  (Yu et al., 2016) |
|  | Dec. in incident T2D European adult males | (Tillin et al., 2015) |
| Isoleucine | Inc. in obese adults | (Felig, Marliss & Cahill, 1969)  (Jeevanandam, Ramias & Schiller, 1991) |
|  | Inc. in T2D adults | (Suhre et al., 2010)  (Wang-Sattler et al., 2012)  (Xu et al., 2013) |
|  | Inc. in T2D adult females | (Menni et al., 2013) |
|  | Inc. in incident T2D adults | (Wang et al., 2011)  (Yamakado et al., 2015) |
|  | Inc. in incident T2D European and S. Asian adult males | (Tillin et al., 2015) |
| Leucine | Inc. in obese adults | (Felig, Marliss & Cahill, 1969)  (Jeevanandam, Ramias & Schiller, 1991)  (Kim et al., 2010)  (Mangge et al., 2016) |
|  | Inc. in T2D adults | (Suhre et al., 2010)  (Xu et al., 2013)  (Villarreal-Pérez et al., 2014) |
|  | Inc. in T2D adult females | (Menni et al., 2013) |
|  | Inc. in incident T2D adults | (Wang et al., 2011)  (Yamakado et al., 2015) |
|  | Inc. in incident T2D European and S. Asian adult males | (Tillin et al., 2015) |
| Lysine | Dec. in obese T2D adult females | (Fiehn et al., 2010) |
| Palmitoylcarnitine (C16) | Inc. in obese adults | (Mihalik et al., 2010) |
|  | Inc. in T2D adults | (Mihalik et al., 2010) |
|  | Inc. in incident T2D adults | (Qiu et al., 2016)  (Sun et al., 2016) |
| Phenylalanine | Inc. in obese adults | (Felig, Marliss & Cahill, 1969)  (Newgard et al., 2009) |
|  | Inc. in T2D adults | (Xu et al., 2013) |
|  | Inc. in incident T2D adults | (Wang et al., 2011)  (Floegel et al., 2014)  (Yamakado et al., 2015)  (Palmer et al., 2015)  (Qiu et al., 2016) |
|  | Inc. in incident T2D S. Asian adult males | (Tillin et al., 2015) |
| Propionylcarnitine (C3) | Inc. in obese adults | (Newgard et al., 2009)  (Kim et al., 2010) |
|  | Inc. in T2D adults | (Mihalik et al., 2010) |
|  | Inc. in incident T2D adults | (Sun et al., 2016) |
| Tryptophan | Dec. in obese adults | (Jeevanandam, Ramias & Schiller, 1991)  (Breum et al., 2003) |
|  | Inc. in obese adults | (Kim et al., 2010) |
| Tryptophan/LNAA | Dec. in obese adults | (Breum et al., 2003) |
|  | Dec. in obese, T2D adults | (Ashley et al., 1985) |
| Tyrosine | Inc. in obese adults | (Felig, Marliss & Cahill, 1969)  (Newgard et al., 2009)  (Kim et al., 2010) |
|  | Inc. in T2D adults | (Villarreal-Pérez et al., 2014) |
|  | Inc. in incident T2D adults | (Wang et al., 2011)  (Yamakado et al., 2015)  (Qiu et al., 2016) |
|  | Inc. in incident T2D European and S. Asian adult males | (Tillin et al., 2015) |
| Valine | Inc. in obese adults | (Felig, Marliss & Cahill, 1969)  (Jeevanandam, Ramias & Schiller, 1991)  (Newgard et al., 2009)  (Kim et al., 2010) |
|  | Inc. in T2D adults | (Suhre et al., 2010)  (Xu et al., 2013)  (Villarreal-Pérez et al., 2014) |
|  | Dec. in T2D adult females | (Menni et al., 2013) |
|  | Inc. in incident T2D adults | (Wang et al., 2011)  (Palmer et al., 2015)  (Yamakado et al., 2015)  (Yu et al., 2016) |
|  | Inc. in incident T2D European and S. Asian adult males | (Tillin et al., 2015) |
