# Supplementary material for: Metabolomic analysis of obesity, metabolic syndrome, and type 2 diabetes: amino acid and acylcarnitine levels change along a spectrum of metabolic wellness
Source: PeerJ. 2018 Aug 31;6:e5410. doi: 10.7717/peerj.5410 (PMC6120443; doi:10.7717/peerj.5410)
Supplement: Table S2 — Only studies reporting data on the contributions of individual plasma/serum amino acids to metabolic unwellness are included in this analysis, resulting in the exclusion of several important studies (Batch et al., 2013; Allam-Ndoul et al., 2016). [file peerj-06-5410-s002.docx]

| **Study** | **Findings (Case vs. Control)** | **Case population studied** | **Metabolic syndrome (MS) criteria** |
| --- | --- | --- | --- |
| (Kamaura et al., 2010) | ↑ Alanine  ↑ Arginine  ↑ Glutamate  ↑ Leucine  ↑ Phenylalanine  ↑ Proline  ↑ Tyrosine  ↑ Valine  ↓ Glycine  ↓ Serine | Adults who met MS criteria | waist circumference ≥ 85 cm for males or > 90 cm for females AND ≥ 2 of the following 3 criteria:  fasting glucose ≥ 110 mg/dL; systolic BP ≥ 130 mm Hg and/or diastolic BP ≥ 85 mm Hg; TG ≥ 150 mg/dL and/or HDL-cholesterol < 40 mg/dL. |
| (Wiklund et al., 2014) | ↑ Alanine  ↑ Isoleucine  ↑ Leucine  ↑ Phenylalanine  ↑ Tyrosine | Premenopausal adult women with BMI 25-40 kg/m^2^ who met MS criteria | at least 3 of the following 5 criteria: waist circumference of ≥ 88 cm; fasting serum TG ≥ 1.7 mmol/L; fasting HDL-cholesterol < 1.30 mmol/L, fasting glucose ≥ 5.6 mmol/L and blood pressure ≥ 130/85 mm Hg. |
| (Yamakado et al., 2015) | ↑ Alanine  ↑ Isoleucine  ↑ Leucine  ↑ Tyrosine  ↓ Serine | Adults who have MS criteria | waist circumference of ≥ 85 cm for males or > 90 cm for females and ≥ 2 of the following 3 criteria: fasting plasma glucose ≥ 110 mg/dL or the use of medication for diabetes; systolic BP ≥ 130 mm Hg and/or diastolic BP ≥ 85 mm Hg or the use of antihypertensive medication; TG ≥ 150 mg/dL and/or HDL-cholesterol < 40 mg/dL or the use of medication for dyslipidemia. |
| (Chen et al., 2015) | Significantly different:  Valine | Metabolically abnormal obese adults with BMI > 25 kg/m^2^ | BMI > 25 kg/m2 and one or more abnormal metabolic indices (fasting blood glucose > 100 md/dL, TG > 150 mg/dL, HDL-cholesterol < 40 mg/dL in males or < 50 mg/dL in females, systolic BP > 130 mm Hg or diastolic BP > 85 mm Hg). |
| (Gao et al., 2016) | ↑ Alpha-aminoadipate  ↑ Isoleucine  ↑ Leucine  ↑ Valine  ↑ Propionylcarnitine | Metabolically unhealthy centrally obese adults | 1) HOMA-IR>4.27 (90th percentile), 2) HDL-cholesterol < 1.03 mM in mmol/L in men and < 1.30 mmol/L in women, 3) fasting blood glucose > 5.6 mmol/L, and 4) waist circumference > 102 cm in men and > 88 cm in women. |
| (Mangge et al., 2016) | ↑ Arginine (OB)  ↑ Phenylalanine (OW and OB)  ↑ Tyrosine (OW and OB)  ↑ Valine (OW and OB)  ↓ Citrulline (LN)  ↓ Glycine (LN)  *See below | Adults with MS | ≥ 2 of the following: (1) impaired fasting glucose ≥ 126 mg/dL; (2) systolic BP ≥ 130 mm Hg or diastolic BP ≥ 85 mm Hg; (3) TG ≥ 150 mg/dL; (4) HDL-cholesterol < 40 mg/dL in males or < 50 mg/dL in females; and insulin resistance (HOMA-IR >5). |
| (Sun et al., 2016) | ↑ C0  ↑ C3, 4, 5, 5:1, 5OH  ↑ C8:1  ↑ C14OH, 16, 18, 18:1, 18:2, 18OH, 20:4  ↓ 3-dehydroxy and 3-dehydroC0  ↓ C7DC  ↓ 3-dehydroxy and 3-dehydroC0  ↓ C7DC  ↓ C12, 12:1 | Adults with MS | Updated ATP III criteria for Asian Americans |

*Parentheses refer to comparisons of metabolically unhealthy to metabolically healthy individuals within a specified BMI category (LN=lean, OW=overweight, OB=obese).
