# Supplementary material for: Metabolomic analysis of obesity, metabolic syndrome, and type 2 diabetes: amino acid and acylcarnitine levels change along a spectrum of metabolic wellness
Source: PeerJ. 2018 Aug 31;6:e5410. doi: 10.7717/peerj.5410 (PMC6120443; doi:10.7717/peerj.5410)
Supplement: Table S3A [file peerj-06-5410-s003.docx]

|  | LMW | OBMW | OBMUW | OBDM |
| --- | --- | --- | --- | --- |
| Storage time-Mean (SD) | 3.2 (0.8) | 3.1 (1.2) | 3.3 (1.2) | 3.9 (1.6) |
