# Supplementary material for: Metabolomic analysis of obesity, metabolic syndrome, and type 2 diabetes: amino acid and acylcarnitine levels change along a spectrum of metabolic wellness
Source: PeerJ. 2018 Aug 31;6:e5410. doi: 10.7717/peerj.5410 (PMC6120443; doi:10.7717/peerj.5410)
Supplement: Table S3B — Numbers of samples by group: LMW (21), OBMW (16), OBMUW (11), OBDM (6). P-values for overall significance (Kruskal-Wallis), Nondiabetic trend (JT trend test), diabetics vs. nondiabetics (Wilcoxon Rank-Sum), and LMW vs. obese nondiabetic (OBNDM, consists of OBMW and OBMUW) subjects (Wilcoxon Rank-Sum). P-values <0.005 were considered significant and are bolded. Statistical tests were conducted as described in Methods ‘Statistical analyses’. [file peerj-06-5410-s004.docx]

| Species | Overall | Obesity Effect  (LMW vs. OBMW, OBMUW) | Metabolic Wellness Trend (Nondiabetic)  (LMW → OBMW → OBMUW) | Diabetic Effect  (LMW, OBMW, OBMUW vs. OBDM) |
| --- | --- | --- | --- | --- |
| Alanine | **0.0008 (↑)** | **0.0005 (↑)** | **0.0005 (↑)** | 0.03**(↑)** |
| Alloisoleucine | **0.002(↑)** | 0.01**(↑)** | **0.001 (↑)** | 0.03**(↑)** |
| Alpha-aminoadipic | **0.0004(↑)** | **0.002 (↑)** | **0.0007 (↑)** | **0.005 (↑)** |
| Cystine | **0.0004(↑)** | **0.001 (↑)** | 0.04**(↑)** | 0.02**(↑)** |
| Isoleucine | **0.0001(↑)** | **0.0005 (↑)** | **0.0003 (↑)** | **0.002 (↑)** |
| Leucine | **<0.0001(↑)** | **0.001 (↑)** | **0.0009 (↑)** | **0.0005 (↑)** |
| Lysine | **0.0003(↑)** | **0.004 (↑)** | **0.003 (↑)** | **0.0009(↑)** |
| Phenylalanine | **0.0003(↑)** | **0.0002 (↑)** | **0.0002 (↑)** | 0.03**(↑)** |
| Propionylcarnitine | **0.003(↑)** | **0.002 (↑)** | **0.001 (↑)** | 0.04**(↑)** |
| Tyrosine | **0.0004(↑)** | **0.0002 (↑)** | **0.0003 (↑)** | 0.03**(↑)** |
| Valine | **0.0005(↑)** | 0.03**(↑)** | **<0.003 (↑)** | **0.001(↑)** |
