# Supplementary material for: Metabolomic analysis of obesity, metabolic syndrome, and type 2 diabetes: amino acid and acylcarnitine levels change along a spectrum of metabolic wellness
Source: PeerJ. 2018 Aug 31;6:e5410. doi: 10.7717/peerj.5410 (PMC6120443; doi:10.7717/peerj.5410)
Supplement: Table S3C — Numbers of samples by group: LMW (21), OBMW (16), OBMUW (11), OBDM (6). P-values for overall significance (Kruskal-Wallis), Nondiabetic trend (JT trend test), diabetics vs. nondiabetics (Wilcoxon Rank-Sum), and LMW vs. obese nondiabetic (OBNDM, consists of OBMW and OBMUW) subjects (Wilcoxon Rank-Sum). P-values <0.005 were considered significant and are bolded. Statistical tests were conducted as described in Table 2. Abbreviations are as follows: GLU, glutamate; GLN, glutamine; HIS, histidine; ILE, isoleucine; LEU, leucine; LYS, lysine; MET, methionine; PHE, phenylalanine; THR, threonine; TRP, tryptophan; TYR, tyrosine; VAL, valine; C3, propionylcarnitine; C5, isovalerylcarnitine. [file peerj-06-5410-s005.docx]

| Sum or Ratio | Overall | Obesity Effect  (LMW vs. OBMW, OBMUW) | Metabolic Wellness Trend (Nondiabetic)  (LMW → OBMW → OBMUW) | Diabetic Effect  (LMW, OBMW, OBMUW vs. OBDM) |
| --- | --- | --- | --- | --- |
| HIS+ILE+LEU+ LYS+ MET+PHE+THR+TRP+ VAL | **0.002(↑)** | 0.01**(↑)** | **0.005 (↑)** | **0.003(↑)** |
| LEU+LYS | **0.0001(↑)** | **0.001 (↑)** | **0.0008 (↑)** | **0.0007 (↑)** |
| $\frac{\mathrm{TRP}}{(TYR+PHE+LEU+ILE+VAL)}$ | **0.0002(↑)** | 0.007**(↑)** | **0.001 (↓)** | **0.0007 (↓)** |
| $\frac{(C3+C5)}{Total carnitine}$ | **0.0009(↑)** | **0.0003(↑)** | **0.0004 (↑)** | 0.05**(↑)** |
