# Supplementary material for: Metabolomic analysis of obesity, metabolic syndrome, and type 2 diabetes: amino acid and acylcarnitine levels change along a spectrum of metabolic wellness
Source: PeerJ. 2018 Aug 31;6:e5410. doi: 10.7717/peerj.5410 (PMC6120443; doi:10.7717/peerj.5410)
Supplement: Table S4 — K-means cluster analysis of the quantitative values of each metabolite does not separate our data into groups based on metabolic wellness by ATP III criteria. All analytes are included except for homocysteine and 3-hydroxybutyrate, which have incomplete data. Each value in the group columns (LMW, OBMW, OBMUW and OBDM) is presented as a count unless otherwise specified. [file peerj-06-5410-s006.docx]

| Cluster | LMW | OBMW | OBMUW | OBDM | Total |
| --- | --- | --- | --- | --- | --- |
| 1 | 8 | 14 | 10 | 10 | 42 |
| 2 | 8 | 4 | 1 | 0 | 13 |
| 3 | 5 | 6 | 7 | 7 | 25 |
| 4 | 3 | 2 | 2 | 3 | 10 |
| Total | 24 | 26 | 20 | 20 | 90 |
