# Supplementary material for: Metabolomic analysis of obesity, metabolic syndrome, and type 2 diabetes: amino acid and acylcarnitine levels change along a spectrum of metabolic wellness
Source: PeerJ. 2018 Aug 31;6:e5410. doi: 10.7717/peerj.5410 (PMC6120443; doi:10.7717/peerj.5410)
Supplement: Table S5A — All amino acids and related compounds were measured in all of the 90 samples except for homocysteine which was measured in all but 5 samples due to lack of volume, yielding the following sample sizes (N = 85), LMW = 24, OBMW = 24, OBMUW = 18, OBDM = 19. Amino acid levels are reported as micromoles/liter and are rounded to 0.1. Values are given as median (Q1, Q3) Bolded values were significantly different upon Kruskal-Wallis testing (p < 0.005). [file peerj-06-5410-s007.docx]

| **Species** | **LMW** | **OBMW** | **OBMUW** | **OBDM** |
| --- | --- | --- | --- | --- |
| Aminobutyrate (ABU) | 18.9 (15.1, 24.4) | 21.1 (16.8, 24.2) | 18.4 (15.3, 27.9) | 21.5 (16.8, 32.7) |
| **Alanine (ALA)** | **352.2 (316.3, 437.4)** | **415.7 (381.6, 486.7)** | **489.7 (446.5, 552.2)** | **492.0 (437.7, 563.2)** |
| **Alloisoleucine (ALLISO)** | **0.9 (0.0, 1.6)** | **1.4 (0.0, 1.7)** | **1.8 (1.6, 2.4)** | **2.2 (1.4, 2.5)** |
| **Alpha-aminoadipate (AAD)** | **0.7 (0.0, 1.0)** | **1.0 (0.9, 1.4)** | **1.3 (1.0,1.7)** | **1.3 (1.1, 1.7)** |
| Anserine (ANSP) | 0 | 0 | 0 | 0 |
| Arginine (ARG) | 94.1 (78.5, 115.5) | 101.7 (87.3, 127.4) | 96.4 (88.2, 145.1) | 104.7 (69.4, 146.5) |
| **Asparagine (ASN)** | **59.8 (55.5, 65.1)** | **56.8 (49.0, 60.5)** | **52.1 (48.5, 61.2)** | **48.0 (45.7, 53.9)** |
| Aspartic acid (ASP) | 17.6 (14.7, 25.8) | 19.6 (15.4, 28.9) | 20.6 (15.8, 26.7) | 19.2 (15.4, 27.5) |
| β-aminoisobutyrate (BABA) | 1.9 (1.4, 2.5) | 1.6 (1.2, 2.1) | 1.5 (1.2,1.8) | 1.4 (1.0, 2.0) |
| β-alanine (BALA) | 7.4 (6.3, 8.3) | 8.4 (7.3, 10.1) | 8.9 (7.5, 10.9) | 8.1 (5.5, 9.3) |
| Carnosine (CARSN) | 3.6 (2.3, 4.7) | 2.9 (2.4, 4.2) | 3.6 (2.8, 5.0) | 3.4 (2.0, 5.2) |
| Citrulline (CIT) | 28.4 (25.7, 32.9) | 28.7 (24.2, 36.4) | 26.6 (24.9, 33.5) | 23.6 (17.7, 34.0) |
| **Cystine (CYS)** | **9.0 (7.0, 10.8)** | **16.0 (10.6, 19.3)** | **14.1 (8.1, 19.5)** | **18.7 (11.6, 21.9)** |
| Ethanolamine (ETN) | 20.2 (17.7, 24.4) | 21.8 (18.1, 26.7) | 20.8 (17.1, 29.7) | 24.2 (19.0, 27.3) |
| GABA (GBA) | 0 (0, 0.3) | 0 (0, 0.2) | 0 (0, 0) | 0 (0, 0.1) |
| Glutamate (GLU) | 223.7 (142.8, 279.2) | 214.7 (164.4, 284.5) | 226.5 (168.3, 288.6) | 239.1 (160.5, 279.3) |
| Glutamine (GLN) | 352.7 (303.1, 445.7) | 383.7 (287.7, 455.3) | 382.2 (327.3, 448.1) | 320.3 (261.9, 390.8) |
| Glycine (GLY) | 283.1 (250.3, 363.6) | 264.9 (214.4, 332.6) | 258.2 (227.0, 277.0) | 252.0 (218.0, 273.3) |
| Homocystine (HCY) | 0 (0, 0) | 0 (0,0) | 0 (0, 0.1) | 0 (0,0) |
| Histidine (HIS) | 91.2 (82.7, 96.4) | 89.6 (81.4, 95.6) | 89.2 (84.9, 100.5) | 87.2 (79.0, 95.7) |
| 1M Histidine (1MHIS) | 5.2 (3.0, 5.6) | 5.0 (3.8, 5.7) | 5.0 (4.2, 6.0) | 4.8 (4.0, 5.9) |
| 3M Histidine (3MHIS) | 15.3 (8.6, 23.8) | 18.0 (13.2, 23.2) | 16.4 (9.0, 23.0) | 12.2 (9.4, 18.1) |
| Homocysteine (HYC) | 9.45 (7.65,10.85) | 9.1 (7.9, 10.4) | 10.3 (8.9, 13.2) | 10.4 (8, 13) |
| Cystathione | 0 | 0 | 0 | 0 |
| Hydroxylysine (HYL) | 0 (0,0.7) | 0 (0, 0.3) | 0 (0, 0.6) | 0.5 (0, 0.7) |
| Hydroxyproline (HYP) | 10.6 (8.0, 13.7) | 13.3 (10.1, 16.7) | 11.0 (9.0, 14.9) | 11.3 (7.9, 17.1) |
| **Isoleucine (ILE)** | **66.1 (63.1, 73.6)** | **76.6 (67.0, 94.5)** | **91.7 (78.6, 102.4)** | **101.5 (88.0, 109.4)** |
| **Leucine (LEU)** | **137.5 (128.2, 154.7)** | **157.4 (140.3, 178.7)** | **173.7 (152.8, 195.4)** | **200.1 (172.7, 213.7)** |
| **Lysine (LYS)** | **197.9 (177.6, 221.4)** | **208.2 (188.2, 228.7)** | **231.6 (206.9, 253.8)** | **231.1 (216.2, 260.8)** |
| Methionine (MET) | 31.3 (26.7, 35.7) | 32.7 (29.6, 37.7) | 33.3 (27.4, 39.7) | 32.8 (28.2, 35.7) |
| Ornithine (ORN) | 59.7 (51.7, 65.0) | 65.5 (55.6, 88.1) | 74.6 (62.5, 86.9) | 62.7 (48.9, 84.7) |
| **Phenylalanine (PHE)** | **65.2 (61.6, 68.9)** | **73.5 (65.8, 79.2)** | **75.5 (70.9, 86.6)** | **77.1 (69.7, 83.8)** |
| Proline (PRO) | 192.7 (170.1, 226.2) | 203.8 (172.7, 256.2) | 225.7 (212.6, 263.3) | 223.0 (185.1, 281.4) |
| Phosphoserine (PSER) | 0 (0, 2.9) | 0 (0, 4.1) | 1.56 (0, 5.9) | 0 (0, 1.3) |
| Sarcosine (SAR) | 0 (0, 1.3) | 0 (0, 0.8) | 0.7 (0, 2.5) | 1.8 (0, 2.3) |
| Serine (SER) | 127.8 (110.7, 144.3) | 127.1 (110.2, 165.8) | 120.5 (102.1, 148.4) | 125.3 (103.7, 154.4) |
| Taurine (TAU) | 164.9 (146.3, 195.0) | 191.0 (156.3, 225.3) | 196.1 (140.3, 219.9) | 180.5 (149.8, 215.0) |
| Threonine (THR) | 145.9 (121.9, 174.5) | 148.1 (125.3, 180.8) | 147.6 (136.0, 163.9) | 127.9 (110.6, 141.2) |
| **Tryptophan (TRP)** | **51.2 (44.2, 56.9)** | **54.0 (49.5, 58.8)** | **55.9 (48.9, 61.2)** | **55.5 (49.7, 58.8)** |
| **Tyrosine (TYR)** | **60.6 (56.6, 67.0)** | **76.9 (70.3, 86.8)** | **82.6 (69.1, 96.2)** | **84.9 (77.3, 93.1)** |
| **Valine (VAL)** | **222.0 (191.1, 254.4)** | **239.6 (219.5, 274.3)** | **284.8 (257.6, 301.9)** | **308.0 (282.8, 341.7)** |
