# Supplementary material for: Metabolomic analysis of obesity, metabolic syndrome, and type 2 diabetes: amino acid and acylcarnitine levels change along a spectrum of metabolic wellness
Source: PeerJ. 2018 Aug 31;6:e5410. doi: 10.7717/peerj.5410 (PMC6120443; doi:10.7717/peerj.5410)
Supplement: Table S5B — Values are given as median (Q1, Q3). Bolded values were significantly different upon Kruskal-Wallis testing (p < 0.005). Acylcarnitine levels are reported as nanomoles/liter, except for free carnitine and acetylcarnitine which are reported as micromoles/liter. Values are rounded to 0.1. [file peerj-06-5410-s008.docx]

| Species | LMW | OBMW | OBMUW | OBDM |
| --- | --- | --- | --- | --- |
| Free Carnitine (C0) | 33.4 (25.5, 36) | 35.5 (28.6,41) | 34.5 (31.0, 45.3) | 30.0 (27, 37.2) |
| Total Carnitine (TC) | 43.1 (35.4, 47.9) | 44.0 (35.8, 48.5) | 45.0 (39.0, 54.8) | 38.0 (36.0, 47.4) |
| Free/Total Carnitine Ratio | 0.8 (0.7, 0.8) | 0.8 (0.8, 0.9) | 0.8 (0.8, 0.8) | 0.8 (0.8, 0.8) |
| Acetylcarnitine (C2) | 10436.5 (7845.3, 13773.3) | 8455.5 (7181.8, 10225.0) | 9834.5 (8079.3, 11667.8) | 11198.0 (8267.0, 12831.0) |
| **Propionylcarnitine (C3)** | **322.8 (249.4, 408.0)** | **471.5 (385.0, 559.9)** | **466.0 (399.1, 569.6)** | **496.5 (399.0, 590.5)** |
| Iso/Butyrylcarnitine (C4) | 98.5 (77.0, 195.0) | 106.0 (94.8, 165.3) | 149.0 (100.0, 184.3) | 174.5 (108.8, 222.3) |
| Isovalerylcarnitine (C5) | 97.0 (71.5, 127.0) | 112.5 (71.0, 144.0) | 109.5 (71.0, 178.0) | 117.5 (98.5, 173.0) |
| Tiglylcarnitine (C5:1) | 6.5 (5.0, 7.8) | 7.5 (5.8, 10.0) | 8.0 (6.0, 11.0) | 8.0 (5.3, 10.8) |
| Hexanolylcarnitine (C6) | 31.0 (21.3, 44.8) | 32.0 (25.5, 47.8) | 37.5 (28.3, 45.0) | 37.5 (29.0, 48.3) |
| Octanoylcarnitine (C8) | 139.0 (86.3, 164.3) | 106.5 (83.8, 151.8) | 116.0 (99.5, 138.8) | 109.5 (94.8, 151.5) |
| Decanoylcarnitine (C10) | 133.5 (82.3, 190.5) | 102.0 (77.5, 165.3) | 112.5 (86.8, 137.0) | 103.0 (87.5, 142.5) |
| Decenoylcarnitine (C10:1) | 142.5 (90.3, 192.8) | 132.5 (92.8, 173.5) | 128.5 (93.3, 161.5) | 132.5 (85.5, 170.3) |
| Dodecanoylcarnitine (C12) | 53.0 (40.0, 67.0) | 42.5 (33.3, 61.0) | 48.5 (35.0, 64.0) | 44.5 (34.5, 57.5) |
| Dodecenoylcarnitine (C12:1) | 19.5 (10.5, 28.5) | 15.0 (8.5, 27.3) | 16.5 (13.0, 25.3) | 15.0 (10.0, 20.8) |
| OH-dodecenoylcarnitine (C12:1-OH) | 2.0 (2.0, 3.0) | 1.5 (1.0, 2.3) | 2.0 (2.0, 3.0) | 2.0 (1.0, 2.8) |
| 3-OH-dodecanoylcarnitine (C12-OH) | 4.5 (3.3, 5.8) | 3.0 (3.0, 5.0) | 4.0 (3.0, 5.0) | 4.0 (3.0, 6.0) |
| Tetradecanoylcarnitine (C14) | 19.5 (17.0, 23.8) | 20.0 (14.0, 24.3) | 21.0 (18.3, 28.8) | 21.5 (17.3, 24.8) |
| Tetradecenoylcarnitine (C14:1) | 37.0 (23.5, 49.3) | 30.0 (19.8, 48.8) | 30.0 (24.3, 40.3) | 29.5 (20.3, 39.8) |
| 3-OH-tetradecenoylcarnitine (C14:1-OH) | 5.5 (4.0, 6.8) | 3.0 (2.0, 5.0) | 4.0 (3.0, 5.8) | 3.5 (3.0, 6.0) |
| Tetradecadienoylcarnitine (C14:2) | 22.5 (12.5, 29.8) | 19.5 (11.8, 27.5) | 16.5 (12.3, 26.0) | 17.5 (10.3, 21.5) |
| 3-OH-tetradecanoylcarnitine (C14-OH) | 4.0 (4.0, 5.0) | 4.0 (3.0, 5.3) | 5.0 (4.0, 6.8) | 5.0 (4.0, 6.8) |
| Hexadecanoylcarnitine (C16) | 99.7 (79.8, 108.3) | 96.0 (89.3, 118.9) | 114.1 (102.7, 141.3) | 117.7 (106.5, 139.0) |
| **Hexadecenoylcarnitine (C16:1)** | **10.5 (6.0, 12.8)** | **8.5 (7.0, 12.8)** | **10.5 (8.0, 12.5)** | **9.5 (7.3, 12.8)** |
| 3-OH-hexadecenoylcarnitine (C16:1-OH) | 1.0 (1.0, 1.8) | 1.0 (1.0, 1.0) | 1.0 (1.0, 1.0) | 1.0 (1.0, 1.0) |
| 3-OH-hexadecanoylcarnitine (C16-OH) | 4.0 (3.0, 5.0) | 4.0 (3.8, 5.0) | 5.0 (4.0, 5.8) | 5.0 (4.0, 6.0) |
| Stearoylcarnitine (C18) | 34.5 (28, 42.5) | 34.0 (27.8, 41.0) | 35.0 (28.3, 43.8) | 31.0 (27.0, 43.8) |
| Oleoylcarnitine (C18:1) | 100.0 (80.3, 118.5) | 90.0 (67.5, 115.5) | 91.0 (72.0, 117.3) | 92.0 (73.5, 118.0) |
| 3-OH-oleoylcarnitine (C18:1-OH) | 1.0 (1.0, 2.0) | 1.0 (1.0, 2.0) | 1.0 (1.0, 1.0) | 1.0 (1.0, 2.0) |
| Linoleoylcarnitine (C18:2) | 43.0 (32.3, 55.0) | 41.0 (32.0, 54.0) | 37.5 (28.8, 51.5) | 36.5 (27.0, 57.5) |
| 3-OH-Linoleoylcarnitine (C18:2-OH) | 1.0 (0.07, 1.0) | 1.0 (0.0, 1.0) | 0.7 (0.0, 1.0) | 1.0 (0.0, 1.0) |
| **Malonylcarnitine (C3DC)** | **88.9 (70.5, 127.0)** | **54.1 (34.7, 71.6)** | **58.8 (49.3, 76.0)** | **66.9 (38.7, 78.7)** |
| Succinylcarnitine (C4DC) | 27.5 (23.0, 37.8) | 29.5 (24.5, 32.8) | 31.0 (26.3, 36.0) | 34.5 (25.0, 39.8) |
| 3-OHbutyrylcarnitine* (C4-OH) | 16.0 (12.0, 25.3) | 13.0 (10.0, 18.5) | 15.0 (10.3, 22.5) | 27.5 (16.5, 35.0) |
| Glutarylcarnitine (C5DC) | 63.0 (45.5, 77.3) | 61.5 (44.5, 69.8) | 53.0 (39.3, 64.5) | 47.0 (38.8, 67.5) |
| OH-Isoval/2Met-3-OH-Butyrylcarnitine(C5-OH) | 23.0 (20.0, 28.5) | 29.0 (23.0, 34.3) | 26.5 (22.3, 33.8) | 29.5 (26.0, 35.0) |

*****3-OHbutyrylcarnitine was specifically quantified for 58 subjects (LMW = 14, OBMW = 20, OBMUW = 11, OBDM = 13). The species quantified in the other subjects could be either 3-OHisobutyrylcarnitine or 3-OHbutyrylcarnitine.
