# Supplementary material for: Metabolomic analysis of obesity, metabolic syndrome, and type 2 diabetes: amino acid and acylcarnitine levels change along a spectrum of metabolic wellness
Source: PeerJ. 2018 Aug 31;6:e5410. doi: 10.7717/peerj.5410 (PMC6120443; doi:10.7717/peerj.5410)
Supplement: Table S5C — Acylcarnitine levels are reported as nanomoles/liter, except for C0 and C2 that are reported as micromoles/liter. Values are rounded to 0.1 and are given as median (Q1, Q3) Bolded values were significantly different upon Kruskal-Wallis testing (p < 0.005). Both glucogenic and ketogenic amino acids are isoleucine, phenylalanine, threonine, tryptophan, and tyrosine. Glucogenic amino acids are alanine, arginine, asparagine, aspartic acid, cystine, glutamate, glutamine, glycine, histidine, methionine, proline, serine, and valine. Abbreviations are as follows: ABU, aminobutyrate; ALA, alanine; ASP, asparagine; CIT, citrulline; GLU, glutamate; GLN, glutamine; HIS, histidine; ILE, isoleucine; LEU, leucine; LYS, lysine; MET, methionine; ORN, ornithine; PHE, phenylalanine; TAU, taurine; THR, threonine; TRP, tryptophan; TYR, tyrosine VAL, valine; C2, acetylcarnitine; C3, propionylcarnitine C4, iso/butyrylcarnitine; C5, isovalerylcarnitine, C6, hexanoylcarnitine; C8, octanoylcarnitine; C10, decanoylcarnitine; C10:1, decenoylcarnitine; C16, hexadecanoylcarnitine (palmitoylcarnitine), C16-OH=3-OH-hexadecenoylcarnitine. [file peerj-06-5410-s009.docx]

| Sum or Ratio | LMW | OBMW | OBMUW | OBDM |
| --- | --- | --- | --- | --- |
| ABU/LEU | 0.1 (0.1, 0.2) | 0.1 (0.1, 0.2) | 0.1 (0.1, 0.1) | 0.1 (0.1, 0.2) |
| ALA/LYS | 1.9 (1.6, 2.2) | 2.1 (1.9, 2.3) | 2.1 (1.8, 2.5) | 2.0 (1.8, 2.3) |
| ALA / (TYR+PHE) | 2.9 (2.6, 3.3) | 2.8 (2.5, 3.2) | 3.0 (2.7, 3.5) | 3.2 (2.8, 3.5) |
| ARG/(ORN+CIT) | 1.1 (0.9, 1.3) | 1.1 (0.9, 1.2) | 1.0 (0.8, 1.5) | 1.3 (0.8, 1.8) |
| **GLU+GLN** | **574.5 (552.8, 617.5)** | **608.1 (567.9, 665.4)** | **617.9 (591.7, 644.8)** | **551.0 (510.4, 583.1)** |
| Both (Gluco+ketogenic-see legend) | 400.3 (363.5, 434.7) | 425.8 (408.7, 484.8) | 455.5 (411.0, 493.6) | 445.3 (409.4, 489.1) |
| Glucogenic AAs (see legend) | 2111.1 (1909.3, 2441.2) | 2228.4 (2046.1, 2460.9) | 2343.4 (2136.8, 2490.0) | 2285.3 (2146.7, 2482.7) |
| **Ketogenic AAs (LEU+LYS)** | **331.7 (307.0, 373.2)** | **362.4 (331.3, 397.3)** | **406.1 (373.0, 447.6)** | **428.0 (391.5, 462.5)** |
| ORN/ARG | 0.6 (0.5, 0.7) | 0.6 (0.5, 0.8) | 0.7 (0.5, 0.9) | 0.6 (0.4, 0.9) |
| ORN/CIT | 2.3 (1.7, 2.5) | 2.3 (1.9, 2.9) | 2.5 (2.0, 3.2) | 2.7 (2.0, 3.1) |
| $\frac{\mathbf{(TAU+CIT+LYS)}}{\mathbf{(ASP+ILE)}}\mathbf{+}\frac{\mathbf{(THR+TYR+HIS)}}{\mathbf{GLU}}$ | **6.4 (5.5, 7.0)** | **5.7 (5.3, 6.1)** | **5.7 (5.2, 6.0)** | **5.0 (4.6, 5.6)** |
| $\frac{\mathbf{TRP}}{\mathbf{(TYR+PHE+LEU+ILE+VAL)}}$ | **0.1 (0.1, 0.1)** | **0.1 (0.1, 0.1)** | **0.1 (0.1, 0.1)** | **0.1 (0.1, 0.1)** |
| $\frac{\mathbf{(VAL+ILE+LEU)}}{\mathbf{(TYR+PHE)}}$ | **3.3 (3.2, 3.7)** | **3.3 (2.9, 3.3)** | **3.5 (3.1, 3.7)** | **3.8 (3.7, 4.2)** |
| $\frac{\mathbf{(C3+C5)}}{\mathbf{Total carnitine}}$ | **10.6 (8.5, 11.7)** | **13.0 (11.2, 15.9)** | **13.1 (10.6, 15.3)** | **15.4 (13.6, 17.8)** |
| C2/Free CN | 0.34 (0.25, 0.39) | 0.24 (0.18, 0.29) | 0.26 (0.19, 0.32) | 0.27 (0.22, 0.33) |
| Free CN/C16 | 0.33 (0.25, 0.44) | 0.33 (0.29, 0.43) | 0.28 (0.24, 0.38) | 0.26 (0.19, 0.35) |
| C16-OH/C16 | 0.04 (0.04, 0.05) | 0.04 (0.04, 0.05) | 0.04 (0.3, 0.05) | 0.04 (0.04, 0.05) |
| C8/C16 | 1.4 (0.9, 1.7) | 1.0 (0.8, 1.5) | 0.9 (0.7, 1.3) | 1.0 (0.8, 1.3) |
| C4/C3 | 0.3 (0.3, 0.5) | 0.3 (0.2, 0.3) | 0.3 (0.2, 0.4) | 0.3 (0.2, 0.5) |
| (C6+C8+C10+C10:1)/Total | 11.3 (5.7, 16.3) | 8.3 (7.4, 11.5) | 9.3 (6.4, 12.8) | 9.7 (7.8, 13.0) |
| C16+/Total | 7.1 (5.3, 9.0) | 6.6 (5.6, 9.2) | 7.1 (5.8, 8.6) | 7.5 (6.0, 9.9) |
